# *De Novo* Multi-Omics Pathway Analysis Designed for Prior Data Independent Inference of Cell Signaling Pathways

**DOI:** 10.1016/j.mcpro.2024.100780

**Published:** 2024-05-03

**Authors:** Katri Vaparanta, Johannes A.M. Merilahti, Veera K. Ojala, Klaus Elenius

**Affiliations:** 1 Turku Bioscience Centre, University of Turku and Åbo Akademi University, Turku, Finland; 2 Medicity Research Laboratories, University of Turku, Turku, Finland; 3 Institute of Biomedicine, University of Turku, Turku, Finland; 4 Department of Oncology, Turku University Hospital, Turku, Finland

**Keywords:** pathway analysis, cell signaling pathways, multi-omics, network modules, TYRO3, cleavage of receptor tyrosine kinases, prior-information independent, association score

## Abstract

New tools for cell signaling pathway inference from multi-omics data that are independent of previous knowledge are needed. Here, we propose a new *de novo* method, the *de novo* multi-omics pathway analysis (DMPA), to model and combine omics data into network modules and pathways. DMPA was validated with published omics data and was found accurate in discovering reported molecular associations in transcriptome, interactome, phosphoproteome, methylome, and metabolomics data, and signaling pathways in multi-omics data. DMPA was benchmarked against module discovery and multi-omics integration methods and outperformed previous methods in module and pathway discovery especially when applied to datasets of relatively low sample sizes. Transcription factor, kinase, subcellular location, and function prediction algorithms were devised for transcriptome, phosphoproteome, and interactome modules and pathways, respectively. To apply DMPA in a biologically relevant context, interactome, phosphoproteome, transcriptome, and proteome data were collected from analyses carried out using melanoma cells to address gamma-secretase cleavage-dependent signaling characteristics of the receptor tyrosine kinase TYRO3. The pathways modeled with DMPA reflected the predicted function and its direction in validation experiments.

## Highlights

• A prior data-independent multi-omics module and pathway inference tool was created.
• The method was able to discover reported associations and outperformed prior art.
• Prediction capabilities were developed to contextualize the results of the method.
• Multi-omics data from cells expressing TYRO3 cleavage variants was acquired.
• The prediction capabilities of the method accurately predicted cell behavior.

## Graphical Abstract

**Figure:**
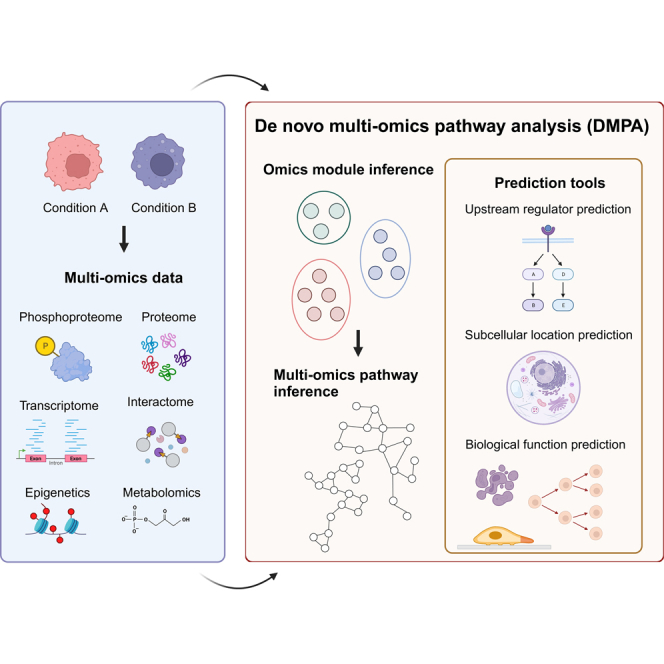

## In Brief

A novel tool, *de novo* multi-omics pathway analysis (DMPA), is introduced for prior data independent and unbiased cell signaling pathway inference from multi-omics data. DMPA was validated with available omics data and outperformed previous module and pathway inference methods, especially with datasets of low sample sizes. DMPA includes upstream regulator, subcellular location, and biological function prediction tools for modules and pathways. DMPA was able to accurately predict cellular behavior from an in-house–acquired multi-omics dataset on TYRO3 signaling.

Several approaches that aim to model cell signaling networks from the abundance or expression information of cell signaling molecules have been developed. These approaches may be divided into two main categories: methods that are reliant on or independent of previous knowledge. The methods that rely on previous knowledge include the classical gene set enrichment analysis (1), its derivatives, and multiple topology-based methods that use previous data as a backbone to infer pathway networks (2). Signaling pathway inference based on previous knowledge, however, poses three major caveats. Firstly, previous knowledge is often incomplete signifying that all identified features are often not covered by the available databases. Secondly, some features are much more researched than others. The unevenness of the available prior knowledge can lead to skewed results in endorsing relationships that have already been uncovered and extensively researched. Thirdly, molecular associations are often context-dependent, indicating that the associations found in a different context may not universally apply.

Robust cell signaling pathway inference methods that are independent of previous knowledge have been generally based on only one measure of molecular association. Correlation, partial correlation, and mutual information have been mainly used as association scores to measure the co-expression or co-abundance of cell signaling molecules (3, 4, 5, 6, 7). Approaches that utilize more than one molecular association measure to derive an integrated score have not been sufficiently explored in the context of prior information-independent methods. Especially approaches that derive and combine multiple association measures from the same level of information have been scarcely utilized. In contrast, approaches that integrate association measures from different levels of information to infer master networks have been successfully applied (8, 9, 10). For instance, correlation measures that represent the co-expression, co-abundance, and genetic co-dependency information have been integrated with random forest regression to infer molecular networks (8, 9).

Regression tree ensemble, Bayesian network, and ODE model based approaches have been developed to solve signaling pathway modeling problems such as gene regulatory networks (11, 12, 13). The transferability of these methods to multi-omics data, however, is largely unexplored. Most accurate renderings of cell signaling networks have been acquired with methods that track changes in time-course data (11, 14). Unfortunately, the cost and feasibility of acquiring time-course data presents a limitation especially in patient samples and in animal models.

The current array of multi-omics integration methods mainly consists of dimensionality reduction and clustering methods that aim to stratify multi-omics data according to disease subtype or patient characteristics and assist in biomarker discovery (15, 16, 17). The module discovery methods such as weighted correlation network analysis (WGCNA) and correlation networks have been expanded and applied to multi-omics data (18, 19). Beyond these efforts, the multi-omics integration methods that aim to learn cell signaling networks seem to rely on previous knowledge (20, 21, 22, 23, 24, 25). A need for a robust scalable benchmark method that can handle diverse omics data, can infer molecular associations from single time point omics data, is independent of previous knowledge and outperforms methods that are based on only one measure of molecular association is evident.

## Experimental Procedures

### The Inference of Network Modules and Pathways in the DMPA

**Fig. 1:**
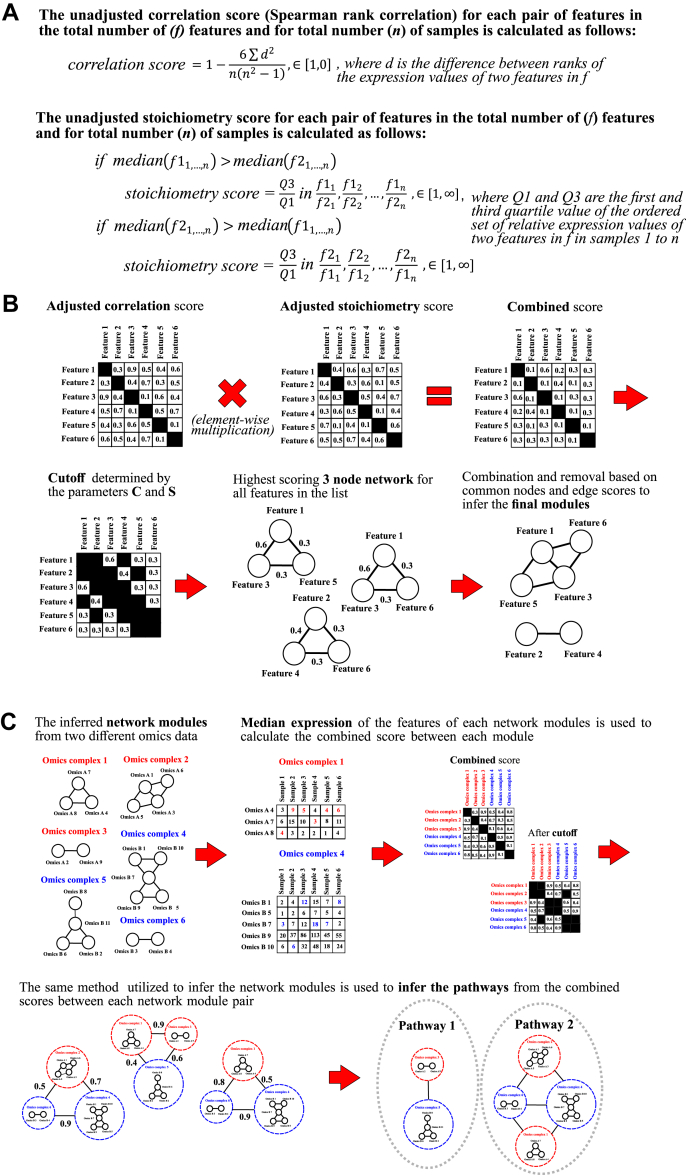
**The underlying concepts of the *de novo* multi-omics pathway analysis.***A*, mathematical formulation of the unadjusted (raw) correlation and stoichiometry scores. *B*, schematic representation of the inference of network modules from the adjusted correlation and stoichiometry scores in the *de novo* multi-omics pathway analysis. *C*, schematic representation of the principles of combining the network modules into multi-omics pathways.

Matlab R2016a (MathWorks) was used as the coding environment. In the module and pathway inference of *de novo* multi-omics pathway analysis (DMPA), the strength of association between two features is determined by the combined score. The combined score is derived from the multiplication of the adjusted correlation and the stoichiometry score. The unadjusted correlation score for all the possible feature pair combinations is calculated with the Spearman correlation. The unadjusted stoichiometry score for all the feature pair combinations in turn is determined by dividing the third quartile value (Q_3_) with the first quartile (Q_1_) value of an ordered set of relative expression values. The mathematical formulations for the unadjusted correlation and stoichiometry scores are presented in Figure 1*A*. The expression values of the features in an omics dataset are supplied to calculate the unadjusted correlation and stoichiometry scores. The unadjusted correlation and stoichiometry scores are ranked by feature and adjusted by the feature-wise rank to derive scores from 1 to 0 in equal increments. The highest unadjusted correlation score and lowest unadjusted stoichiometry score for the feature pair including a specific feature are assigned 1 as the adjusted score. Two versions of the stoichiometry score were devised for non-zero–inflated and zero-inflated data. In the non-zero–inflated version of the stoichiometry score, relative expression data is considered only from samples in which the expression value for both features is non-zero. In the zero-inflated version of the stoichiometry score (weighted stoichiometry score, parameter 4 in supplemental Fig. S1), the stoichiometry score is punished (inflated) if the expression value for one feature is zero. The stoichiometry score, in turn, is rewarded (deflated) if the expression value for both features is zero.

Cut-off parameters C and S were devised to reduce computational time and to filter out false positives to only consider associations with combined scores higher than a certain threshold (supplemental Fig. S1). The cut-off parameter C is determined by the minimum number of associations considered per feature in the analysis. The cut-off parameter S, in turn, is determined by the number of features in the dataset that are estimated to not have any true association. The cut-off parameters C and S are used to find a threshold for the combined score that allows at least C number of feature pairs for S number of features to be included for further analysis. After selecting the feature pairs with a combined score above the threshold determined by the C and S parameters, a filtering step ensures that no feature pair with a significant discrepancy in their feature-wise combined score is included in further analysis. The threshold for the filtering is determined by a parameter (parameter 10 in supplemental Fig. S1) that sets the threshold for the filtering based on the expected score difference between the set number of features.

The network module inference proceeds by finding maximally scoring three feature cliques (sum of the combined scores between all three features) for each remaining feature. The three feature modules are combined and trimmed based on common features and module scores (average of the combined score for all features in a module) as described in the following order: (1) modules with two common features are combined. (2) Modules with only one common feature are combined if the size of both modules is under or equal to the set parameter 7 value and the module score is higher or equal to the value set by the parameter 8 (default value 0.66, supplemental Fig. S1) indicating a strong association. The parameter for this joining (parameter 7 in supplemental Fig. S1) can be set if modules of different size are preferred. (3) Modules with two common features are again combined. (4) Features of the remaining three feature modules are removed if some of the features are already present in larger modules. (5) The remaining small modules are joined if they still have a common feature. This step can be omitted and the threshold for combined size of the joined modules can be adjusted (parameter 9 in supplemental Fig. S1). The inference allows for a feature to be a member of more than one module.

The same strategy is used for the combination of the network modules into pathways as for the initial module inference with few modifications. (1) The sample-wise median values of all features in a network module are used as expression values for network modules. (2) The absolute value of the Spearman correlation is used as the unadjusted correlation score to allow equal combination of upregulated and downregulated modules (parameter 3 in supplemental Fig. S1). The pseudocode of DMPA can be found in the Supplementary Materials. The default settings of DMPA were used for the analysis of the TYRO3 signaling pathways.

### Validation of Network Modules and Their Combination to Pathways

The reported protein–protein interactions, kinase substrate phosphosite relationships, transcription factor target gene relationships, methylation trait relationships, and metabolic reactions were acquired from the STRING, PhosphoSitePlus, ChEA3, and Rhea databases, respectively (26, 27, 28, 29, 30, 31). The annotated signaling pathways were acquired from PathwayCommons (32). The validation interactome, phosphoproteome, transcription, and metabolomics data were acquired from the publications of Batth *et al.* and Hein *et al.* and from ArchS4 and MetaboLights database, respectively (33, 34, 35, 36) (supplemental Table S14). The methylation data and multi-omics data for the validation of the network module combination to pathways was acquired from the LinkedOmics database (37), where a combination of either proteomics, transcriptomics, methylome, phosphoproteomics, protein array, glycosylation, or acetylation data were analyzed and combined. Two different validation scores were derived for network modules and pathways, respectively. The sum of all protein–protein interactions, kinase substrate phosphorylation site relationships, transcription factor target gene relationships, metabolites in the same metabolic reaction, or methylated regions associated with the same trait in the inferred network modules was used as the validation score for interactome, phosphoproteome, transcriptome, metabolome, and methylome network module validation, respectively. The sum of co-occurrences of two features from two different network modules in the same pathway annotation for inferred pathways was used as a validation score for the combination of network modules into pathways. To validate the network modules, the validation score from the inferred network modules was compared to the sum of all protein–protein interactions, kinase substrate phosphorylation site relationships, transcription factor target gene relationships, metabolites in the same metabolic reaction, or methylated regions associated with the same trait in randomly modeled network modules of same size to derive an empirical probability density function from 1000 rounds of simulation. To validate the combination of network modules into pathways, the validation score from the inferred pathways was compared to the sum of all co-occurrences of two features in two different randomly modeled and combined modules of same size in the same pathway annotation to derive an empirical probability density function from 1000 rounds of simulation. An Epanechnikov kernel (38) was used to fit the sum of all co-occurrences from randomized modules or pathways into an empirical probability density function. The corresponding *p*-values were drawn from the empirical cumulative distribution function. The relative distance of the validation score from the median of the empirical probability distribution was calculated by subtracting the median value from the validation score and by dividing the remainder with the median value. To assess the effect of different association scores on the accuracy of the network modules and pathways, the modules were inferred with either the correlation score, stoichiometry score, or the combined score and combined with either correlation score, stoichiometry score, or the combined score. The conservation of the different scores was assessed in each data type by deriving the median score of the inferred network modules or pathways in datasets different from the one used for the initial inference.

### Benchmarking

The transcriptome datasets and multi-omics datasets utilized in DMPA validation were additionally used for benchmarking (supplemental Table S14). The R implementations of WGCNA (3), low-order partial correlation (LOPC) (39), GENIE3 (13), and Transkingdom Network Analysis (TransNet) (40) were used according to the developers' instructions. WGCNA was additionally used to analyze multi-omics data as in the MiBiOmics application (18). The power for the WGCNA analysis was set based on the scale-free topology model fit and mean connectivity as instructed by the WGCNA documentation. The genes predicted to be controlled by the same upstream gene in GENIE3 were considered as part of the same module. The validation score and statistical significance of the validation score were estimated for modules derived with WGCNA and GENIE3 as for the network modules of DMPA. Since LOPC only returns network edges, a separate validation strategy to compare the performance of LOPC and DMPA was developed. The number of reported transcription factor target gene relationships in the network edges returned by LOPC as determined by the ENCODE database (29) was calculated and used as a validation score. The validation score was compared to the sum of all transcription factor target gene relationships in randomly modeled modules of same size to derive an empirical probability density function from 1000 rounds of simulation. An Epanechnikov kernel (38) was used to fit the simulated data into the empirical probability density function. The corresponding *p*-values and relative distance from the median of the randomized distribution were estimated from the empirical cumulative distribution function. To compare DMPA against LOPC, the number of reported transcription factor target gene relationships in the final edges in the network modules as suggested by the remaining combined scores after module joining and trimming were considered as network edges. The validation score and the empirical probability distribution were similarly estimated for the networks suggested by DMPA as for the networks suggested by LOPC. The validation score for the pathways suggested by the TransNet and multi-omics WGCNA analysis were estimated as those for the DMPA. The clusters connected by the connected nodes by the TransNet analysis and the modules connected by a significant correlation of their eigenvectors by WGCNA were considered a pathway. A version of the pathway validation method where only the connections between different modules were randomized was developed and applied to TransNet, multi-omics WGCNA, and DMPA.

### Data Simulation

Simulation strategy adapted from Zuo *et al.* (5) (2014) was utilized to simulate network modules. The modules $X1=\left\{x_{1},x_{2},x_{3},x_{4},x_{5}\right\}$ and $X2=\left\{x_{6},x_{7},x_{8},x_{9},x_{10}\right\}$ were defined as:

$$x_{1}=s_{1}+e_{1}$$

$$x_{2}=\lambda_{1}{\times x}_{1}+e_{2}$$

$$x_{3}={\alpha_{1}\times x}_{2}+e_{3}$$

$$x_{4}={\alpha_{2}\times x}_{3}+e_{4}$$

$$x_{5}={\alpha_{3}\times x}_{4}+{\lambda_{2}\times x_{1}+e}_{5}$$

$$x_{6}={\mu_{1}\times x}_{10}+e_{6}$$

$$x_{7}={\alpha_{4}\times x}_{6}+e_{7}$$

$$x_{8}={\alpha_{5}\times x}_{7}+e_{8}$$

$$x_{9}={\alpha_{6}\times x}_{8}+{\mu_{2}\times x_{10}+e}_{9}$$

$$x_{10}=s_{2}+e_{10}$$

In the simulated data for parameter S and C sensitivity analyses $s_{1},s_{2}∼N\left(Y_{1},1\right)$ and $e_{1}-e_{10}∼\;N\left(1,Y_{1}÷10\right)$, where $Y_{1}\;∼\;Z\in \left[2,30\right]$ and $\lambda_{1},\lambda_{2},\alpha_{1}-\alpha_{6},\mu_{1,}\mu_{2}$ were set to 1. In the simulated data following the negative binomial distribution $s_{1},s_{2}∼NB\left(Y_{2},0.05\right)$ and $e_{1}-e_{10}\;∼\;NB\left(Y_{2},0.5\right)$, where $Y_{2}\;∼\;Z\in \left[2,15\right]$ and $\lambda_{1},\lambda_{2},\alpha_{1}-\alpha_{6},\mu_{1,}\mu_{2}$ were set to 1. In the simulated data following the beta distribution $s_{1},s_{2}∼B\left(Y_{3},2\right)$ and $e_{1}-e_{10}∼\;B\left(Y_{3},50\right)$, where $Y_{3}∼\;Z\in \left[2,10\right]$ and $\lambda_{1},\lambda_{2},\alpha_{1}-\alpha_{6},\mu_{1,}\mu_{2}$ were set to 1. The datapoints with no true module assignment were simulated as $s_{3}-e_{11}$, where $s_{3}\;∼\;Z\in \left[1,35\right]$ and $e_{11}∼\;N\left(0,1\right)$ or $s_{3}∼NB\left(Y_{4},0.05\right)$ and $e_{11}∼NB\left(Y_{4},0.5\right)$ and $Y_{4}∼\;Z\in \left[2,15\right]$ or $s_{3}\;∼\;B\left(Y_{4},3\right)$ and $e_{11}\;∼\;B\left(Y_{4},50\right)$ and $Y_{4}∼\;Z\in \left[2,10\right]$, respectively. Fifty samples were simulated for parameter S and C sensitivity analyses. In the simulated data used to estimate data dimensionality requirements of DMPA $s_{1},s_{2}∼N\left(Y_{1},1\right)$ and $e_{1}-e_{10}∼\;N\left(Y_{1}÷5,Y_{1}÷50\right)$, where $Y_{1}\;∼\;Z\in \left[2,10\right]$.

### Cell Culture and Transfections

WM-266-4 human melanoma cancer cells and HEK293T cells were cultured in Dulbecco’s modified Eagle’s medium (Life Technologies) supplemented with 10% (weight/vol) fetal calf serum (Promocell). For the transfection of WM-266-4 cells, jetPRIME (Polyplus-transfection) transfection reagent was used according to the manufacturer’s protocol. To produce lentiviral particles, the HEK293T cells were transfected with the lentiviral packaging and shRNA plasmids with FuGENE6 (Roche) according to the manufacturer’s instructions.

### ADAM10 and ADAM17 Cleavage Site Prediction

The cleavage site prediction method was modified from a previously published transcription factor–binding prediction method (41). The positional relative frequencies of the amino acid sequences in putative a disintegrin and metalloprotease 10 and 17 (ADAM10 and ADAM17) cleavage sites were calculated from the results of two published peptide screens (42, 43). The cleavage sites for ADAM10 and ADAM17 were predicted with a sliding window analysis. The probability for an ADAM cleavage motif based on the positional relative frequencies of amino acid residues with a 0^th^ order Markov chain was calculated from sequence windows of 10 residues. The probability for no ADAM cleavage motif was calculated from the same sequence windows with 0^th^ order Markov chain using the relative frequencies of amino acid residues in proteins as determined by Uniprot (44) as positional frequencies. The sequence windows with at least two times higher probability for an ADAM cleavage motif than no ADAM cleavage motif were considered probable cleavage sites. Additional constraint for the cleavage motifs to reside within 35 amino acids from the transmembrane domain was added, due to known ectodomain size constraints of gamma-secretase substrates (45). The method was validated by ensuring its capability in finding published cleavage sites in the peptides described in Goth *et al.* (46), 2015. P408/L409 and A418/G419 of TYRO3 were predicted to serve as cleavage sites with highest predicted relative cleavage site probabilities for both ADAM10 and ADAM17.

### Plasmids and Cloning

The pDONR223-TYRO3ΔGS vector including a TYRO3 insert with a mutated gamma-secretase cleavage site (I449A; TYRO3 ΔGS) has been previously described (Merilahti *et al.*, 2017). Constructs encoding TYRO3 with mutated ADAM cleavage sites (P408A/L409P/G419P triple mutation; TYRO3 ΔADAM) were generated using synthetic DNA fragments (Integrated DNA Technologies) and assembled into pDONR223-based TYRO3 Gateway entry plasmid (Addgene kit #1000000014) (47, 48) using NEBuilder HiFi DNA Assembly Master Mix (New England Biolabs). WT TYRO3, TYRO3 ΔGS, and TYRO3 ΔADAM constructs were cloned from pDONR223 plasmids into pEZY-myc-his (Addgene #18791) (49) and pMAX-DEST (Addgene #37631) (50) expression plasmids using Gateway Cloning Technology with LR Clonase II Enzyme Mix (Life Technologies) to allow expression of C-terminally Myc-his-tagged and V5-tagged proteins, respectively. Mutations were verified by sequencing.

For the production of lentiviral particles carrying shRNAs, the following plasmids were used: pLKO.1-puromycin plasmid (containing the shRNA) (Sigma-Aldrich), pRSV-Rev, pMDLg/pRRE, and pMD2.G (a gift from Didier Trono; Addgene plasmids #12253, #12251, and #12259). Restriction digestion with EcoRI was used to verify the integrity of the commercial plasmids.

### Antibodies

Anti-TYRO3 (5585), anti-V5 (13202), anti-phospho-STAT3 (8862), and anti-STAT3 (9972) antibodies were purchased from Cell Signaling Technology. Anti-actin (MA1-744) antibodies were purchased from Thermo Fisher Scientific. Anti-β-tubulin (T7816) and anti-phosphotyrosine (4G10; 5-321) antibodies were purchased from Sigma-Aldrich.

### Immunoprecipitation and Western Analysis

For immunoprecipitation experiments, WM-266-4 vector control cells or cells expressing Myc-tagged TYRO3 constructs were incubated in serum-free conditions overnight before lysis into lysis buffer (0.1% Triton X-100, 10 mM Tris–Cl pH 7.4, 150 mM NaCl including Pierce protease and phosphatase inhibitor mini tablet (Thermo Fisher Scientific)). The cell lysates were pre-cleared with 50 μl Pierce protein G magnetic beads (Thermo Fisher Scientific) at 4 °C for 1 h and subjected to affinity enrichment with Pierce anti-c-Myc magnetic beads (Thermo Fisher Scientific) at 4 °C overnight. The beads were washed five times with 5 TBS-T buffer (125 mM Tris, 750 mM NaCl, 0.25% Tween-20) and once with ultrapure water. The proteins were denatured and eluted from the beads by incubation at 95 °C for 10 min in SDS-PAGE loading buffer.

For Western analyses, cell lysates were prepared as above. The cells were treated with 5 μM of GSI IX (Calbiochem) for 4 hours before lysis where indicated. The lysates were run on SDS-PAGE gels and the separated proteins were transferred to nitrocellulose membranes. The membranes were incubated with indicated primary antibodies and IRDye-conjugated secondary antibodies (LI-COR) to label the proteins of interest. The IR-signals were detected with the Odyssey CLx imaging system (LI-COR).

### Generation of Stable TYRO3 Knock-Down Cell Lines

For stable downregulation of TYRO3, TYRO3-targeted lentiviral shRNA plasmid TRCN0000231528 (CCGGTTGGTATCTCAGGTCTGAATCCTCGAGGATTCAGACCTGAGATACCAATTTTTG) (MISSION, Sigma-Aldrich) was used. The control lentiviral shRNA plasmid (Addgene plasmid #1864) was a kind gift from David Sabatini (51). Third generation lentiviral packaging system (Addgene) was used in HEK293T cells to produce shRNA-carrying lentiviruses. Growth medium was changed every 24 h post-transfection. Virus-containing medium was collected after 48 and 72 h, filtered, and tittered. The virus-containing medium was used to infect WM-266-4 cells at a multiplicity of infection of 2 in the presence of 8 μg/ml Polybrene (Sigma-Aldrich). Cells were maintained in the presence of 1 μg/ml puromycin (Sigma-Aldrich) to select the cells that stably express the lentiviral shRNA plasmids.

### RNA-Sequencing

The WM-266-4 cell transfectants were starved in serum-free medium overnight and lysed. Total RNA was extracted using NucleoSpin RNA Plus kit (Macherey-Nagel).

The quality of the RNA samples was ensured using Advanced Analytical Fragment Analyzer (Agilent). The sequencing library was created with 300 ng of sample with TruSeq Stranded mRNA HT Kit (Illumina) and indexed with IDT for Illumina TruSeq RNA UD Indices according to manufacturer’s protocol. Genome-wide strand-specific RNA-seq was performed at Turku Bioscience Centre sequencing core with Illumina HiSeq3000 using 75 bp paired-end reading.

### Mass Spectrometry Sample Preparation

The WM-266-4 cell transfectants were lysed in mass spectrometry lysis buffer (6 M guanidine hydrochloride, 100 mM Tris–HCl pH 8.5, 5 mM Tris(2-carboxyethyl)phosphine, 10 mM chloroacetamide) or affinity enrichment lysis buffer (70 mM octyl-β-D-glucopyranoside, 25 mM Tris–HCl pH 7.5, 150 mM NaCl, Pierce protease and phosphatase inhibitor mini tablet). The lysates were centrifuged at 20000*g* for 6 min, and the supernatants were collected. The protein concentration of the lysates was measured with Bradford protein assay (Bio-Rad) before proceeding to affinity enrichment or to protein digestion.

### Affinity Enrichment of Mass Spectrometry Samples

The WM-266-4 cell transfectants were subjected to protein crosslinking with 2 mM DTBP for 10 min. The crosslinking reaction was quenched by incubation with 50 mM Tris–HCl pH 7.5 for 15 min before cell lysis. Equal amounts of WM-266-4 cell lysates were pre-cleared with Pierce protein G magnetic beads (Thermo Fisher Scientific) at 4 °C for 1 h and subjected to affinity enrichment with HisPur Ni-NTA magnetic beads (Thermo Fisher Scientific) at 4 °C overnight. The beads were washed five times with 5 TBS-T buffer (125 mM Tris, 750 mM NaCl, 0.25% Tween-20) and once with ultrapure water. The proteins were denatured, alkylated, and eluted from the beads at 95 °C for 10 min in elution buffer (6 M guanidine hydrochloride, 100 mM Tris–HCl pH 8.5, 10 mM Tris(2-carboxyethyl)phosphine, 10 mM chloroacetamide).

### Protein Digestion

Proteins enriched in affinity enrichment or purified cell lysates were digested with lys-C (enzyme/protein ratio 1:100) for 1 h at 37 °C. The samples were diluted 1:10 with 50 mM NH_4_HCO_3_ and digested with trypsin (enzyme/protein ratio 1:100) at 37 °C overnight.

### Sample Desalting

Sep-Pak tC18 96-well plate (Waters) was activated with 100% methanol and conditioned with 0.1% TFA in 80% acetonitrile (Thermo Fisher Scientific). Digested peptides were acidified to a pH 3 with TFA and desalted with the activated and conditioned Sep-Pak tC18 96-well plate. The peptides were washed with 0.1% TFA and eluted with 0.1% formic acid in 50% acetonitrile. Samples were dried in Hetovac vacuum centrifuge (Heto Lab Equipment) and stored dry in −20 °C until analysis with mass spectrometer.

### Phosphopeptide Enrichment

Phosphopeptides were enriched from desalted and dried peptides using Pierce high-select TiO_2_ phosphopeptide enrichment kit (Thermo Fisher Scientific) according to the manufacturer’s instructions. Following elution, phosphopeptides were dried with Hetovac vacuum centrifuge and stored as dry in −20 °C until analysis with mass spectrometer.

### Mass Spectrometry

Dried peptide samples were resuspended in 0.1% formic acid and sample concentrations were measured with Nanodrop 1000 (Thermo Fisher Scientific). Equal amounts of samples were analyzed on an Easy-nLC 1000 coupled to an Orbitrap Fusion Lumos instrument (Thermo Fisher Scientific) at Turku Bioscience Centre Proteomics Core Services. Peptides were loaded on in-house packed 100 μm × 2 cm precolumn packed with ReproSil-Pur 5 μm 200 Å C18-AQ beads (Dr Maisch) using 0.1% formic acid in water (buffer A) and separated by reverse phase chromatography on a 75 μm × 15 cm analytical column packed with ReproSil-Pur 5 μm 200 Å C18-AQ beads (Dr Maisch). All separations were performed using a 60 min gradient ranging from 8% buffer B (80% acetonitrile in 0.1% formic acid) to 21% in buffer B in 28 min and to 36% buffer B in 22 min and ramped to 100% buffer B in 5 min at flow rate of 300 nl/min. The washout followed at 100% buffer B for 5 min.

All MS spectra were acquired on the orbitrap mass analyzer and stored in centroid mode. For data-dependent acquisition experiments, full MS scans were acquired from 300 to 1600 m/z at 120,000 resolution with fill target of 7E5 ions and maximum injection time of 50 ms. The most abundant ions on the full MS scan were selected for fragmentation using 1.6 m/z precursor isolation window and beam-type collisional-activation dissociation (HCD) with 30% normalized collision energy for a cycle time of 3 s. MS/MS spectra were collected at 15,000 resolution with a fill target of 5E4 ions and maximum injection time of 100 ms. Fragmented precursors were dynamically excluded from selection for 35 s.

### Protein Identification and Quantification

MS/MS spectra were searched with MetaMorpheus (version 0.0.303; https://smith-chem-wisc.github.io/MetaMorpheus/) (52) against human proteome containing known post-translational modifications (downloaded from Uniprot on 19.2.2019). The search database contained 20,645 protein sequences including 490 contaminant sequences added by MetaMorpheus software. The mass spectrometry files were calibrated and possible posttranslational modifications were searched with G-post-translational modification (PTM)-D search in MetaMorpheus. Following parameters were used for the PTMs search: cysteine carbamidomethylation and methionine oxidation were set as constant and variable modifications, respectively and common biological, metal, or artifact PTMs were incorporated into the search database with G-PTM-D task. Peptides and proteins were identified with final search using the augmented search database with incorporated G-PTM-D–based modifications. Precursor mass tolerance was set to 5 ppm and product mass tolerance was set to 20 ppm. Peptide and protein quantification was performed with the FlashLFQ algorithm (53). Peptides were accepted with search engine score above 5. Search results were filtered to a 1% false discovery rate at peptide-spectrum match and protein level. For both protein and peptide data files, only proteins or peptides identified in target database and proteins present in at least three of the samples were retained for downstream analyses.

### RNAseq Data Analysis

The RNAseq reads were quality checked with FastQC (Babraham Bioinformatics), quality and adapter trimmed with PRINSEQ (54) and Trimmomatic (55), and pseudoaligned with kallisto v 0.46.0 (56) to human transcriptome Ensembl v96 (57) to retrieve transcripts per million values. The batch effect between experiment 1 and experiments 2 and 3 was corrected with batchelor (58) and the transcripts per million values were normalized by library size. Differential expression between the vector control sample and samples representing the different variants of TYRO3 was analyzed with DeSeq2 (59). Transcripts with fold change over 1.5 and false discovery rate-adjusted *p*-value lower or equal than 0.05 were selected for further analysis. The transcripts significantly different between the vector control sample and all of the samples representing the TYRO3 variants (WT, ΔADAM, or ΔGS) were considered to reflect the signaling mediated by the full-length TYRO3 receptor. The transcripts significantly different between the vector control sample and the sample representing WT TYRO3, but not between the vector control sample and the samples representing ΔADAM or ΔGS variant of TYRO3, were considered to reflect the signaling mediated by the soluble intracellular domain (ICD) of TYRO3.

### Mass Spectrometry Data Analysis

The interactome, proteome, and phosphoproteome data were normalized to the sum of intensities of all detected proteins in the sample. A probability density function was fitted with Epanechnikov kernel to median normalized intensities of different treatments to estimate the *p*-value for differential expression from the cumulative density function. Proteins with fold change over 1.5 and false discovery rate-adjusted *p*-value lower or equal to 0.05 were selected for further analysis. The co-precipitating proteins, proteins, and phosphorylated residues significantly different between the vector control sample and all of the samples representing the TYRO3 variants (WT, ΔADAM, or ΔGS) were considered to reflect the signaling mediated by the full-length TYRO3 receptor. The proteins significantly different between the sample representing WT TYRO3 and the vector control sample as well as between the sample representing WT TYRO3 and samples representing the ΔADAM or ΔGS variant of TYRO3 were considered to reflect the signaling mediated by the soluble ICD of TYRO3.

### Immunofluorescence and Confocal Microscopy

To detect ectopically expressed V5-tagged TYRO3 constructs, WM-266-4 transfectants were cultured on coverslips in serum-free conditions overnight. The cells were fixed with 4% paraformaldehyde and permeabilized with 0.1% Triton X-100. Cells were stained with anti-V5 (13202; Cell Signaling Technologies) and AlexaFluor 555 goat anti-rabbit (Molecular Probes). After labeling the nuclei with 4′,6-diamidino-2-phenylindole (Sigma-Aldrich), the cells were mounted with Mowiol 40-88 (Sigma-Aldrich). Images were acquired with Zeiss LSM 880 confocal microscope (Zeiss). Morphological analysis were carried out from confocal slices in plane with the plasma membrane using MorphoLibJ v 1.4.2.1 (60) plugin in Image J. EzColocalization plugin (61) in ImageJ (version 1.53c) was used to analyze colocalization from confocal slices taken from the middle of the nucleus. The colocalization of V5-signals with 4′,6-diamidino-2-phenylindole was calculated using Pearson correlation coefficient (62) to measure the nuclear localization of TYRO3.

### Live-Cell Imaging of Cell Growth

The WM-266-4 cell transfectants were plated in serum-free medium at 30,000 cells/24-well. The wells were imaged every 2 h for 120 h and cell confluence was measured with Incucyte ZOOM (Sartorius).

### Real-Time Cell Adhesion Assay

The WM-266-4 cell transfectants were detached using 5 mM EDTA 6 to 12 h after transfection. The cells were plated at 15,000 cells/well onto fibronectin-coated (5 μg/cm^2^) xCELLigence E-plate (Agilent) wells in serum-free medium. Real-time cell impedance was measured with the xCELLigence RTCA analyzer (Agilent) for 24 h. The resulting cell index values were normalized to the number of cells remaining in the wells after 24 h.

### Information Visualization and Statistics

For statistical analysis, the GraphPad Prism software v 9.02 (GraphPad Software; https://www.graphpad.com/features) and Matlab 2016a (MathWorks; https://se.mathworks.com/products/matlab.html) were utilized. The details of the statistical testing are described in figure legends. All datasets were tested for equality of variance and normality, and parametric or nonparametric testing was used accordingly. Heatmaps were generated with Morpheus (https://software.broadinstitute.org/morpheus). The visualizations of the inferred network modules and pathways were created with Cytoscape (Version 3.8.2) (63) and Affinity Designer (Version 19.0; Serif).

### Experimental Design and Statistical Rationale

Three biological replicate experiments and three technical replicates per experiment were analyzed in the proteomics studies. To examine the differential interactome, proteome, and phosphoproteome between the WT and cleavage-resistant variants of TYRO3, the proteome, interactome, and phosphoproteome of the cell expressing the WT TYRO3 (n = 3) was compared to the proteome, interactome, and phosphoproteome of both cleavage resistant variants of TYRO3 (n = 6). Likewise, the proteome of the full-length TYRO3 was determined by comparing the proteome, interactome, and phosphoproteome of the no TYRO3 control (n = 3) to the proteome, interactome, and phosphoproteome of all TYRO3 variants (n = 9). Non-parametric testing was utilized. Statistical significance was reached at an alpha level of 0.05 with non-parametric testing with unequal sample size per analyzed group.

## Results

### The Underlying Concepts of DMPA

We set out to develop a new robust *de novo* method to infer network modules from different omics data and to combine the modules into multi-omics pathways. Literature search of previous reports of the key features of omics data predicting molecular associations produced the following observations: First, multiple sources indicate the ability of correlation of expression values to predict molecular association to a certain extent (64, 65). Secondly, the findings from an extensive human interactome study indicate that conserved stoichiometry between the expression values of two co-precipitated proteins can be predictive of protein–protein interaction networks (36). From the grounds of these two concepts, a combined score that serves as a metric for both correlation and conserved stoichiometry was devised to reflect the strength of association between two features. The suitability of the conserved stoichiometry as an association score was additionally explored since it is unknown whether stable between-sample stoichiometry between the expression values of two features reflects the strength of association in diverse omics data. In Figure 1*A*, the mathematical formulation for the unadjusted correlation and stoichiometry score is presented. The unadjusted correlation score is based on the nonparametric Spearman correlation. The unadjusted stoichiometry score in turn is derived from the division of the third quartile and first quartile value in an ordered set of the relative expression values of two features. The unadjusted correlation and stoichiometry scores for each possible feature pair for one feature are ranked and adjusted to values between 1 and 0 by rank to allow for a linear scale. The highest correlation score and lowest stoichiometry score for the feature are assigned as 1 and receive the highest adjusted score. The combined score is expressed as a simple non-weighted feature-wise multiplication of the adjusted correlation and stoichiometry scores. Nonparametric formulation was chosen to desensitize the inference to technical variation and outlier values.

To reduce computational demand (O(n^2^) for run time) and false positives, a cut-off for the combined score was devised to consider only strong associations. In the current version of DMPA, the cut-off threshold is controlled by two parameters that reflect the number of associations considered for the features in the data (parameter C) and the predicted number of features with no true association in the data (parameter S) (supplemental Fig. S1).

The inference of network modules from the combined score after the cutoff was designed to follow the nearest neighbor principle, which assumes that a high association score between three features is more predictive of true association than a high score between only two features. The inference method is outlined in Figure 1*B* and utilizes the combined scores above the cut-off threshold to find maximal scoring 3-feature cliques. These 3-feature cliques are consequently combined to find larger modules and trimmed to remove features that are present in a larger module. The principles of the combination and trimming are outlined in more detail in the Experimental Procedures section.

The combination of the network modules to pathways was designed to follow a similar principle to the initial inference of the network modules (Fig. 1*C*). A median expression value of all the features in a module in each sample is used as a representation of the expression values of a module. The combined score for each module pair is calculated based on these values. To allow equal combination of down- and up-regulated modules, an absolute value is taken from the Spearman correlation to equally weigh positive and negative correlations. The modules are then combined further into pathways based on the combined scores above the after-cut-off threshold with the same rules for combining and trimming the maximal scoring 3 feature cliques that were used in the initial network module inference.

The *de novo* multi-omics pathway analysis is available both as a matlab code and executables in Mendeley data: https://data.mendeley.com/datasets/m3zggn6xx9/draft?a=71c29dac-714e-497e-8109-5c324ac43ac3. Installation instructions, directions for use, and a thorough description of all the parameters of DMPA are provided in the accompanying documentation. The DMPA software is open source and available under the CC-BY license.

### Validation of the Network Modules Inferred with DMPA

The performance of the derived network module inference method was assessed with published interactome (36), phosphoproteome (34), and transcriptome (33) data (supplemental Table S14). While molecular associations are context-dependent, certain associations are more frequently reproduced in different contexts. Therefore, a level of conservation is expected from a score that would accurately reflect the strength of molecular association. The conservation of the correlation, stoichiometry, and combined scores were examined by inferring modules from the expression or abundance values of proteins, phosphorylation sites, or transcripts by utilizing the different molecular association scores. The median value of the scores of the inferred modules was examined in another dataset apart from the one used for the initial module inference. A median score above 0.5, the median score expected if the scores would be randomly assigned, indicated conservation.

**Fig. 2:**
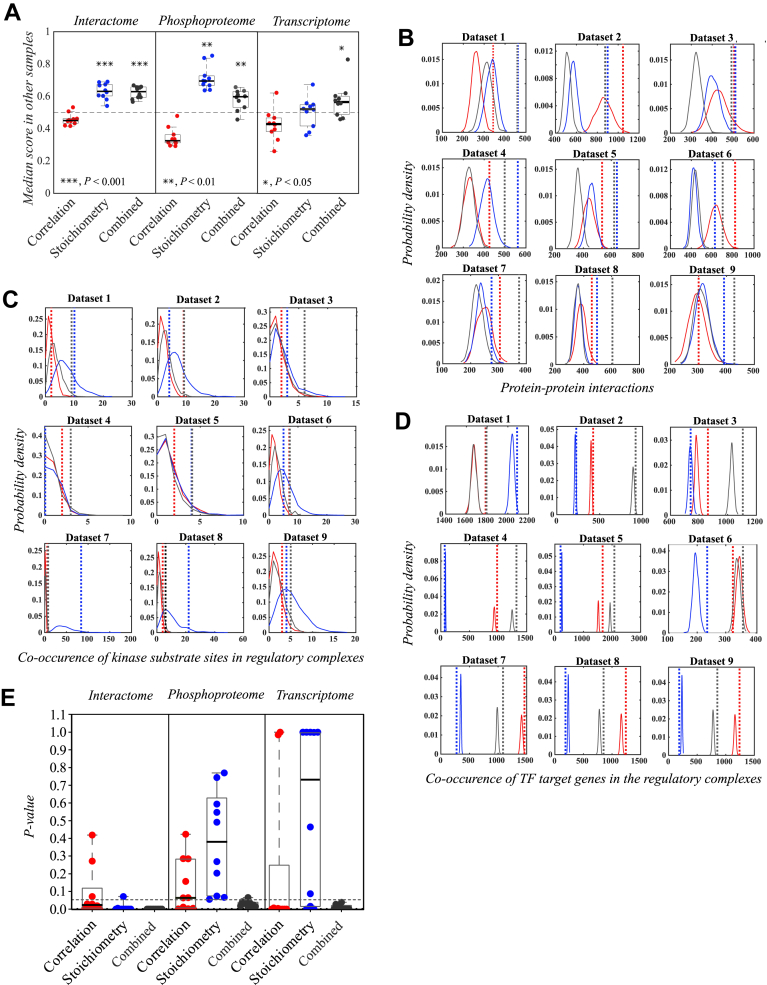
**Validation of network modules inferred with the DMPA.***A*, the conservation of correlation, stoichiometry, and combined score in the interactome, phosphoproteome, and transcriptome data. One dot represents the median score between all features of all modeled modules of a dataset that was not utilized for the initial network module inference. The *dashed line* represents the median score of randomized modules. For statistical testing, two-tailed one sample *t* test against the theoretical median value of 0.5 was utilized. *B*–*E*, empirical probability densities simulated by randomizing the features of the inferred network modules into modules of the same size and the corresponding *p*-values (*E*) for the validation scores of the modules inferred with DMPA. The network modules were inferred from either interactome (*B* and *E*), phosphoproteome (*C* and *E*), or transcriptome data (*D* and *E*) by utilizing either the correlation (Corr), the stoichiometry (Stoi), or the combined score (Comb). The sum of co-occurrences of protein–protein interactions as determined by the STRING database, substrates phosphorylated by the same kinase as determined by the PhosphoSitePlus database, or genes regulated by the same transcription factor as determined by the ENCODE database in the network modules were used to determine the empirical probability density and the validation score for the inferred modules. The empirical probability densities are visualized with color-coded solid lines and the validation scores for the inferred modules in color-coded *dashed lines*. One dot in the boxplot represents one *p*-value for one dataset, the box the interquartile range, and the *horizontal line* the median value. The datasets were acquired from published omics data. *Red*: correlation score; *blue*: stoichiometry score; *gray*: combined score. DMPA, *de novo* multi-omics pathway analysis.

In all examined omics data (supplemental Table S14), the combined score was statistically significantly conserved, while the stoichiometry score was significantly conserved only in the mass spectrometry–derived interactome and phosphoproteome data (Fig. 2*A*). The correlation score in turn was below the 0.5 threshold value in all tested omics data, indicating that the correlation score alone is least likely to predict molecular association (Fig. 2*A*). The conservation of the combined score was similarly examined in methylome, metabolomics, and protein acetylation data. The combined score was discovered to be significantly conserved also in these omics data (supplemental Fig. S4*A*).

To validate that the network modules reflect previously reported molecular associations, a method to quantify the number of reported associations in the network modules was devised. Reported protein–protein interactions, kinase substrate phosphorylation site relationships, and transcription factor target gene annotations were downloaded from STRING (26), PhosphoSitePlus (27), and ENCODE (29) databases, respectively. The sum of all co-occurrences of proteins reported to interact, substrate sites reported to be phosphorylated by the same kinase, or transcripts reported to be the target gene for the same transcription factor inside the network modules was used as a validation score. The validation score was calculated from the network modules inferred with correlation, stoichiometry, and combined scores to compare the effect of the different scores on the accuracy of DMPA. The validation score quantified from the network modules was compared to the sum of all co-occurences of proteins reported to interact, substrate sites reported to be phosphorylated by the same kinase, or transcripts reported to be the target gene for the same transcription factor of randomly formulated network modules of the same size to calculate the probability that a similar validation score would be acquired randomly. The probability density of the sum of all co-occurences of these randomized network modules are indicated with a solid line and the validation scores for the network modules modeled with the different scores with dashed lines in the Figure 2, *B*–*D*. The corresponding *p*-values drawn from the cumulative probability densities of the randomized network modules for the validation scores of the network modules modeled with different scores are visualized in Figure 2*E*.

The network modules modeled with the combined scores consistently reflected reported molecular associations in all interactome, phosphoproteome, and transcriptome data. In interactome data, the co-occurrence of two proteins in the same network module predicted a protein–protein interaction between the two proteins more often than random assignment to network modules of the same size. Additionally, the median STRING score, which reflects the confidence of the interaction based on the amount and type of evidence on the protein–protein interaction, was consistently higher in the network modules inferred with the combined score than in the randomized network modules (supplemental Fig. S2). In the phosphoproteome data, the assignment to network modules modeled with the combined score indicated that the two phosphorylation sites were controlled by the same kinase more often than by random assignment. In the transcriptome data, in turn, two transcripts in the same network module modeled with the combined score were more likely to be the target gene for the same transcription factor than randomly chosen transcripts. This effect was similarly observed in transcription factor target gene annotations acquired from REMAP and Literature libraries from the ChEA3 database (28) (supplemental Fig. S3). The network modules inferred with only the correlation or stoichiometry score varied in their ability to find reported molecular associations. The network modules inferred with stoichiometry score were sufficient, although not as consistent as the network modules inferred with the combined score, in reflecting reported protein–protein interactions in interactome data. However, the network modules inferred with stoichiometry score failed to reproduce reported molecular associations in phosphoproteome and transcriptome data. The network modules inferred with the correlation score were in 8 out of 10 cases able to capture known transcription factor target gene relationships in the transcriptome data but were insufficient in finding reported protein–protein interactions and kinase substrate relationships in the interactome and phosphoproteome data, respectively. The ability of the network modules inferred with the combined score to predict reported molecular associations from available metabolomics and methylation data was additionally examined. The network modules inferred with the combined score were able to predict metabolites involved in the same metabolic reaction in metabolomics data and methylated chromatin regions associated with the same trait in methylome data (supplemental Fig. S4*B*). All in all, the network modules inferred with the combined score consistently captured reported molecular associations in all tested omics data, while the modules inferred with other scores were found lacking in reproducibility and performance across omics data types.

### Validation of Network Module Combination to Pathways by DMPA

**Fig. 3:**
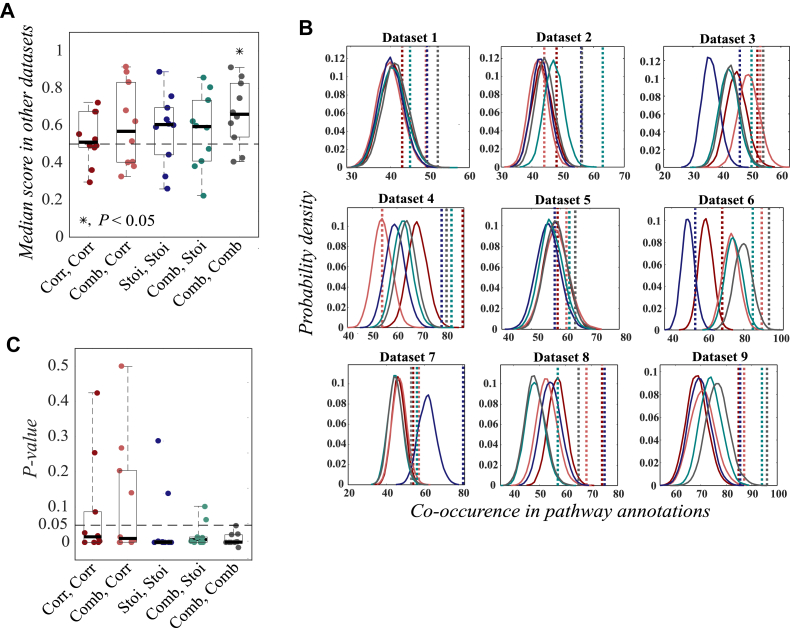
**Validation of network module combination to pathways with DMPA.***A*, the conservation of correlation, stoichiometry, and combined score in modules inferred with either correlation (Corr), stoichiometry (Stoi), or combined (Comb) score and integrated into pathways with either correlation, stoichiometry, or combined score. One dot in the boxplot represents the median score between all modules of all inferred pathways of a dataset that was not initially utilized for the pathway inference. The *horizontal line* represents the median value and the *dashed line* the median score from randomized pathways. For statistical testing, two-tailed one sample *t* test against the theoretical median value 0.5 was utilized. *B* and *C*, empirical probability densities simulated by randomizing the inferred modules into pathways of the same size and the corresponding *p*-values (*C*) for the validation scores of the pathways inferred with DMPA. Acetylation, methylation, proteomics, glycoproteomics, phosphoproteomics, transcriptomics, and protein array data from LinkedOmics was utilized. The sum of all co-occurrences of signaling molecules in separate network modules reported to be part of the same signaling pathway and combined into the same pathway by DMPA was used as a validation score for the inferred pathways. The PathwayCommons database was used as a source for the pathway annotations. One dot in the boxplot represents a *p*-value for one dataset and the line the median value. The datasets were acquired from published multi-omics data. The empirical probability densities are visualized with color-coded *solid lines* and the validation score for the inferred pathways in color-coded *dashed lines*. Corr, Corr (*dark red*): modules inferred with correlation score, pathways integrated with correlation score. Comb, Corr (*light red*): modules inferred with correlation score, pathways integrated with combined score. Stoi, Stoi (*dark blue*): modules inferred with stoichiometry score, pathways integrated with stoichiometry score. Comb, Stoi (*turquoise*): modules inferred with stoichiometry score, pathways integrated with combined score. Comb, Comb (*dark gray*): modules inferred with combined score, pathways integrated with combined score. DMPA, *de novo* multi-omics pathway analysis.

Since signaling pathways are a combination of molecular associations, they are similarly expected to be partially conserved. The conservation of the pathways inferred with DMPA was assessed. Network modules were inferred from a multi-omics dataset with either correlation, stoichiometry, or combined score and integrated into pathways with either correlation, stoichiometry, or combined score. The median score of the inferred pathways was assessed from a multi-omics dataset that was not utilized for the initial pathway inference. Multi-omics data from normal and cancer tissue samples from the LinkedOmics (37) database was utilized. The conservation was tested both between different paired cancer and normal samples and between different cancer stages. Utilizing the combined score for both network module and pathway inference was the only tested combination that demonstrated statistically significant conservation (Fig. 3*A*). Utilizing the stoichiometry or correlation score for either network module or pathway inference in turn produced pathways that were not conserved across datasets.

To ensure that DMPA is also able to combine the inferred network modules into pathways that reproduce experimentally validated signaling pathways, a validation method was devised. The sum of all co-occurrences of signaling molecules in separate network modules reported to be part of the same signaling pathway and combined into the same pathway by DMPA was used as a validation score for the inferred pathways. The reference signaling pathways were downloaded from the PathwayCommons database (32). Validation scores calculated from randomly simulated and combined pathways of the same size were used to derive probability densities to estimate the statistical significance for the validation scores derived from the pathways inferred with DMPA. The probability densities derived from randomly formulated pathways are indicated with solid curves and the validation scores for the pathways combined and inferred with different scores with dashed lines in Figure 3*B*. The corresponding *p*-values drawn from the cumulative probability densities of the randomized pathways for the validation scores of the multi-omics pathways inferred with different scores are visualized in Figure 3*C*. The pathways where the network modules were inferred and integrated with the combined score consistently reproduced associations represented in reported pathway annotations. The second-best performers were the versions of DMPA where the network modules were inferred with either the stoichiometry score or the combined score and integrated into pathways with the stoichiometry score. The correlation score-based versions of DMPA had the worst performance. Taken together, these results strongly imply that the DMPA can find molecular associations and pathways that reflect previously observed cell signaling pathways.

### Validation of the Design Decisions of DMPA

To explore the validity of the design decisions of DMPA, the effect of different design decisions of DMPA on the accuracy of DMPA was examined as in Figure 2, *B*–*E*. Since the *p*-values in many cases approached zero, the relative distance of the validation score from the median value of the randomized distribution was used as a measure instead of the *p*-value like in Figure 2, *B*–*E*. First, the effect of the choice to rank and adjust the stoichiometry and correlation score on the number of reported molecular associations in the inferred modules was assessed. In both interactome and transcriptome data, the choice to rank and adjust the stoichiometry and correlation score (combination, ranked) increased the number of reported associations in the inferred molecular associations against the version of the combination score where the unadjusted correlation score and the inverse of the unadjusted stoichiometry score were multiplied (combination, raw) (supplemental Fig. S5*A*). In phosphoproteome data, the choice to rank and adjust the correlation and stoichiometry had no significant effect. Second, the effect of the design choice to find the maximally scoring three-feature modules for each feature in the data instead of a linear approach, where for each feature only the maximally scoring feature is selected, was examined. For both interactome and phosphoproteome data searching the maximally scoring three-feature modules instead of the single maximally scoring feature increased the number of previously discovered molecular associations in the inferred feature relationships (supplemental Fig. S5*B*). For transcriptome data, the design choice had no significant effect. Taken together, the results imply that the design decisions of DMPA have mostly a positive effect or a neutral effect depending on the examined omics data on discovering true positives in the inferred molecular associations. To synthetize, the effect of adhering to both design decisions increased the number of reported associations in the inferred molecular associations for all tested omics data types.

### Sensitivity Analyses of the Parameters of DMPA

To assess the sensitivity of DMPA to parameter choice, sensitivity analyses were conducted. First, the effect of the parameter choice on the parameters that control the size of the network modules (parameters 7 and 9, supplemental Fig. S1) was examined with available interactome data (supplemental Fig. S6). Settings where the module joining controlled by these parameters were not allowed were tested (supplemental Fig. S6*A*). The change in these parameters affected the size and number of network modules but had no negative effect on the number of reported associations in the inferred modules against randomly modeled modules (supplemental Fig. S6). This suggests that the parameters can be safely adjusted if larger or smaller modules are desired from the inference method.

Second, the effect of the parameter choice of the parameters C and S that control the cut-off threshold of the combined score was assessed in both available omics and simulated data (Figure 1*B*, parameters 5–6 in supplemental Fig. S1). To examine the effect of the parameter S choice on the number of reported molecular associations in the inferred modules and on the algorithm performance, published transcriptome data was analyzed with different S parameter values. Different S parameter values had a significant effect on the run time but no adverse effect on the number of reported molecular associations in the inferred modules (supplemental Fig. S7). To test the effect of the S parameter on the true positive and false positive rate, DMPA was applied to normally distributed, negative binomially distributed, and beta-distributed simulated data (supplemental Figs. S8–S10). DMPA was able to accurately discover the true modules among the randomly modeled data from the simulated data. Some sensitivity to the parameter S choice, however, was detected. Low parameter S values increased the likelihood of false positives and disproportionately high parameter S values increased the likelihood of false negatives in the final inference. Features with a true module assignment were, however, never misassigned by DMPA. The false positives detected with low parameter S values originated from the simulated pool that had no true module assignment. The raw unadjusted correlation and stoichiometry scores of the features that were not assigned into network modules were examined and a significant decrease in the maximum raw correlation and inverted raw stoichiometry score was discovered for the features that had no true module assignment (supplemental Fig. S11*A*). To this end, a script to suggest values for the parameter S was devised to help the user to correctly set the parameter S value (supplemental Fig. S11*B*, suggest parameter values for module and pathway inference function of DMPA). The script finds the maximum correlation and inverted stoichiometry scores for each feature and plots the density function that is fitted into these values. The maximum correlation and inverted stoichiometry scores for the features included in the analysis after cut-off are indicated at each parameter S value.

The effect of the parameter C choice on the true and false positive rate of DMPA was also examined in simulated datasets with different proportions of non-associated features in the simulated datasets (supplemental Fig. S12). For datasets for more than 300 features, the parameter C value was discovered to always be optimal as 1. For datasets with less than 300 features, the optimal C parameter value was based on the proportion of non-associated features in the dataset. To this end, the suggested parameter values for module and pathway inference function was expanded to include recommendations for parameter C values to help the user set the optimal parameter value for the dimensionality of and proportion of nonassociated features in their omics datasets.

Third, the sensitivity of DMPA to the parameter that controls the zero-inflated version of the analysis was assessed. Zero-inflated interactome data and non-zero-inflated transcriptome data was analyzed both with the zero-inflated (weighted stoichiometry score, parameter 4 in supplemental Fig. S1) and non-zero-inflated (non-weighted stoichiometry score) version of DMPA, and the number on reported molecular associations in the inferred modules against randomly modeled modules of the same size was estimated. In the zero-inflated interactome data (over 30% missing values), the zero-inflated version of the analysis significantly increased the number of reported associations in the inferred modules (supplemental Fig. S13*A*). In contrast, in the non-zero-inflated transcriptome data (less than 25% missing values), the zero-inflated version discovered fewer reported associations than the non-zero-inflated version (supplemental Fig. S13*B*). In conclusion, the choice of zero-inflated and non-zero-inflated version of the analysis has a significant effect on the accuracy of DMPA and should be set based on the percentage of missing values in the dataset.

### Dataset Dimensionality Requirements of DMPA

To examine how the dimensionality of the omics datasets impacts the performance of DMPA, extensive sensitivity analyses with simulated datasets were conducted (supplemental Fig. S14). The false positive and true positive rates were assessed from datasets with varying feature set and sample sizes and proportions of non-associated features in the datasets. DMPA analyses with different C and S parameter values were additionally performed. Consistent performance of DMPA was observed with sample sizes 10 or higher. With lower sample sizes, DMPA had varied performance, and the nature of the dataset significantly affected the accuracy of DMPA. DMPA benefitted from higher parameter S values when datasets with low sample sizes were analyzed. Due to this observation, “the suggest parameters for module and pathway inference” function was designed to offer parameter value recommendations based on the dimensionality of the omics dataset. Datasets with feature set sizes larger than 200 and sample sizes lower than 10 are not currently recommended to be analyzed with DMPA. Similarly, consistent performance of DMPA with datasets with feature set sizes lower than 200 and sample sizes lower than 8 cannot be guaranteed.

### DMPA Outperforms Previous Approaches

**Fig. 4:**
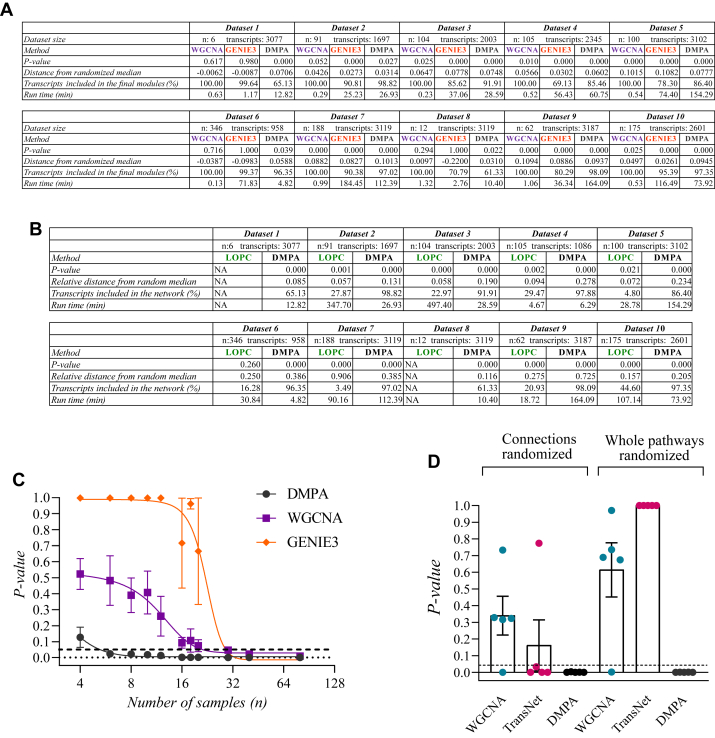
**The performance of DMPA compared to previous methods.***A*, the performance of DMPA against WGCNA and GENIE3 in module discovery. The statistical significance of the number of reported transcription factor target gene relationships and percentage of transcripts included in the inferred modules was assessed in addition to the runtime. Transcriptome data from ArchS4 database was used for benchmarking. *B*, the performance of DMPA against LOPC in network discovery. The statistical significance of the number of reported transcription factor target gene relationships and percentage of transcripts included in the inferred networks was assessed. Transcriptome data from ArchS4 database was used for the benchmarking. NA: LOPC unable to infer a network from the dataset due to the high-dimensionality problem. *C*, the performance of DMPA against WGCNA and GENIE3 in module discovery with transcriptome datasets of different sample sizes. The statistical significance of the number of reported transcription factor target gene relationships in the inferred modules was assessed. Mean ± SEM. *D*, the performance of DMPA against TransNet and WGCNA in pathway discovery. Multi-omics data was analyzed with both methods and the number of reported associations in pathway annotations from the pathway Commons database in the inferred pathways was assessed. The statistical significance of the number of the reported associations in the inferred pathways was determined with two different statistical methods, where either only the connections between the modules or the whole pathways were randomized. Mean ± SEM. DMPA, *de novo* multi-omics pathway analysis; LOPC, low-order partial correlation; WGCNA, weighted correlation network analysis.

To assess the performance of DMPA, DMPA was benchmarked against relevant module discovery and multi-omics integration methods. Transcriptome datasets were chosen for the benchmarking of module discovery methods since they allowed the comparison against regulatory network inference methods that are specifically designed for gene expression data. WGCNA (3), LOPC (5), and the DREAM4 challenge winning regulatory network inference method GENIE3 (13) were selected for comparison. Since LOPC returns network edges instead of modules, a separate validation strategy was devised to benchmark against LOPC which is detailed in the Experimental Procedures section. For GENIE3, a module was defined as the genes predicted to be controlled by the same upstream gene. The number of reported associations in the inferred modules and networks against randomly modeled modules and networks was assessed from the transcriptome modules and networks inferred with WGCNA, LOPC, GENIE3, and DMPA as in Figure 2, *B*–*E*. Although all WGCNA, LOPC, and GENIE3 were able to discover significantly more reported molecular associations in the inferred modules than random assignment, DMPA was the most consistent performer across datasets and in most cases discovered more reported molecular associations than the other methods as indicated by the lower *p*-values and higher relative distance from the random median value (Fig. 4, *A* and *B*). Strikingly, a clear difference in the performance of DMPA and the other tested methods could be seen with datasets with low sample sizes. DMPA seemed to display more discriminatory behavior (smaller coverage) but higher accuracy (lower *p*-value and higher relative distance from random median) with datasets with low sample sizes. LOPC in turn was unable to analyze the datasets with low sample sizes due to the high dimensionality, n ≪ *p* problem (66) (NA in Fig. 4*B*).

To further assess the performance of DMPA and the other module discovery methods with low sample sizes, the module discovery methods were run with transcriptome datasets with different sample sizes. The number of reported molecular associations in the inferred modules against the randomly modeled modules were examined. DMPA required datasets with only six samples (3 control and 3 treatment samples) to discover reported molecular associations while datasets with over 30 samples were required by WGCNA and GENIE3 (Fig. 4*C*). We attempted to analyze zero-inflated interactome data with WGCNA, GENIE3, and LOPC, but the current R implementations of these approaches were unable to handle the zero-inflated interactome data. DMPA, in contrast, has the advantage of performing without the imputation of the missing values for the zero-inflated data. These results imply that DMPA is more flexible, has less limitations, and can discover more reported associations than the tested module and network discovery methods.

DMPA was additionally benchmarked against multi-omics integration methods that are independent of prior knowledge. DMPA was benchmarked against the closest discovered analog methods, TransNet (40) and the multi-omics version of WGCNA (18). TransNet aims to discover causal links between two different omics datasets by first modeling the omics datasets into correlation networks. TransNet then proceeds by assigning dense subnetworks into modules, and finally utilizes correlation to find the connecting nodes between the modules of the omics datasets. The multi-omics version of WGCNA in turn applies correlation and hierarchical clustering to determine modules from each omics layer. The modules are consecutively connected by the correlation of their eigenvectors.

Published multi-omics datasets were analyzed with TransNet, WGCNA, and DMPA, and the capacity of the inferred pathways to represent reported signaling pathways was estimated as in Figure 3, *B* and *C*. The ability to connect inferred modules into signaling pathways was also estimated by a version of the validation method utilized in Figure 3, *B* and *C* where only the connections but not the modules themselves were randomized. As per its aim, TransNet was able to significantly discover connections between the modules that represented reported cell signaling pathways, but the modules themselves were not representative of cell signaling pathways as suggested by the lack of a significant *p*-value from the validation method that also randomized the modules (Fig. 4*D*). The multi-omics version of WGCNA, in turn, was only able to discover reported signaling pathways and connections when transcriptome and proteome data was analyzed (Fig. 4*D*). DMPA, however, was able to achieve a significant *p*-value with both versions of the validation method for all tested datasets indicating that the pathways inferred with DMPA are closer to reported cell signaling pathways than the outputs of TransNet and the multi-omics version of WGCNA.

### Case Study: DMPA of Cleaved TYRO3 Signaling in Melanoma Cells

To demonstrate the performance of DMPA with a freshly acquired multi-omics dataset, an in-house multi-omics dataset from the cleavage-dependent signaling of the receptor tyrosine kinase TYRO3 was acquired. To this end, a stable TYRO3 knock-down WM-266-4 cell line was created and the cells were transfected with WT and cleavage-resistant variants of TYRO3 (ΔGS, ΔADAM) or control vector (supplemental Fig. S15*A*). The ΔGS variant, resistant to proteolytic cleavage by the gamma-secretase complex, has been described before (67). The ΔADAM variant, resistant to cleavage by ADAM, was created by predicting the ADAM17 of ADAM10 cleavage sites of TYRO3 with a 0th order Markov chain and mutating the key residues in the predicted cleavage sites. The ability of the ΔADAM variant to still act as a functional receptor was validated by examining its cleavage (supplemental Fig. S15*B*), autophosphorylation (supplemental Fig. S15*C*), and ability to activate a substrate with Western blot analyses (supplemental Fig. S15*D*). The expected effect of the mutations in the ΔADAM variant on the subcellular location of TYRO3 was examined with immunofluorescence (supplemental Fig. S15*E*). The analyses strongly suggested that the ΔADAM variant was indeed a functional receptor that was more resistant to cleavage than the WT receptor. Interactome, phosphoproteome, proteome, and transcriptome data were acquired from the cells expressing the WT or cleavage-resistant variants of TYRO3 or the control vector (supplemental Fig. S15*F*). A differential expression analysis was conducted to discover the differential interactome, phosphoproteome, transcriptome, and proteome associated with the full-length or the cleaved ICD of TYRO3 (supplemental Figs. S16–S21, and supplemental Table S6–S9).

The differentially regulated signaling events associated with the expression of full-length TYRO3 or the cleaved TYRO3 ICD were analyzed with DMPA first to extract network modules. The DMPA discovered 24 previously undescribed interactome, 110 phosphoproteome, 46 transcriptome, and 130 proteome modules that associated with the expression of full-length TYRO3 (supplemental Fig. S22, and supplemental Table S2). The expression of the cleaved TYRO3 ICD in turn was associated with 53 new interactome, 84 phosphoproteome, 45 transcriptome, and 136 proteome modules (supplemental Fig. S23, and supplemental Table S2). The inferred modules were further combined with the DMPA into pathways, resulting in a total of 51 and 46 unique signaling pathways associating with the signaling stimulated by the full-length and the released ICD of TYRO3, respectively (supplemental Table S1).

### Enrichment Analyses for Modules and Pathways Inferred With the DMPA

To contextualize the function of the modules and pathways inferred with DMPA, an enrichment analysis to predict the upstream transcription factor, upstream kinase, subcellular location, and the biological process for each transcriptome, phosphoproteome, and interactome module or pathway, respectively, was devised (see Experimental Procedures for details). The predicted upstream transcription factors, kinases, and subcellular locations for the transcriptome, phosphoproteome, and interactome modules are provided in supplemental Tables S3–S5. To ensure the validity of the upstream transcription factor and kinase predictions, the predicted transcription factors and kinases were compared to those reported to be regulated by TYRO3. Several transcription factors reported to be regulated by TYRO3 were predicted to regulate the inferred transcriptome modules, such as STAT3 (68), MYC (69, 70), and MITF (71) (supplemental Table S3). Furthermore, the most common transcription factor predicted to regulate the transcriptome modules associated with TYRO3 signaling was MYC, with the respective gene amplified in the WM-266-4 cell background (72). Similarly, several kinases reported to be associated with TYRO3, such as AKT (73, 74), mTOR (73, 75), GSK3 (74, 75), MEK1 (75), PKC (75), and CAMK2 (75), were predicted to regulate the phosphoproteome modules associated with full-length and cleaved ICD of TYRO3 (supplemental Table S5). The predicted subcellular localizations of the interactome modules of full-length and the cleaved ICD of TYRO3 (supplemental Table S4), in turn, were compared to the reported localization difference between the WT and the ΔGS and ΔADAM variants of TYRO3 (supplemental Fig. S15*E*) (67) to ensure the validity of the predicted subcellular locations. The cleavage-resistant variants of TYRO3 have lower nuclear localization compared to the WT TYRO3 (supplemental Fig. S15*E*) (67). Due to this observation the interacting proteins of the cleaved ICD of TYRO3 are expected to be localized more in the nucleus and less in the plasma membrane than those of the full-length TYRO3. The differences in the predicted localizations of the interactome modules of full-length and cleaved ICD of TYRO3 correlated with the difference in the subcellular localization of full-length and cleaved ICD of TYRO3 (supplemental Table S4). Thirteen out of the fifty four (24%) cleaved ICD interactome complexes were predicted to localize into the nucleus, while nuclear localization was predicted for only 2 out of the 21 (8%) interactome modules of the full-length TYRO3 (*p* = 0.032 against frequency of nuclear assignment in the interactome complexes of cleaved TYRO3 ICD). Moreover, 8 out of the 21 (33%) interactome complexes of full-length TYRO3, but only 5 out of the 54 (9%) interactome modules of the cleaved ICD of TYRO3, were predicted to localize into the plasma membrane (*p* < 0.0001 against frequency of plasma membrane assignment in the interactome modules of full-length TYRO3). Both the interactome modules of full-length TYRO3 (5 out of 21; 20%) and the interactome modules of TYRO3 ICD (11 out of 54, 20%) were equally predicted to be localized in the cytoskeletal structures (*p* = 0.0025 for full-length TYRO3 and *p* < 0.0001 for TYRO3 ICD against frequency of cytoskeleton assignment in randomly modeled interactome modules).

**Fig. 5:**
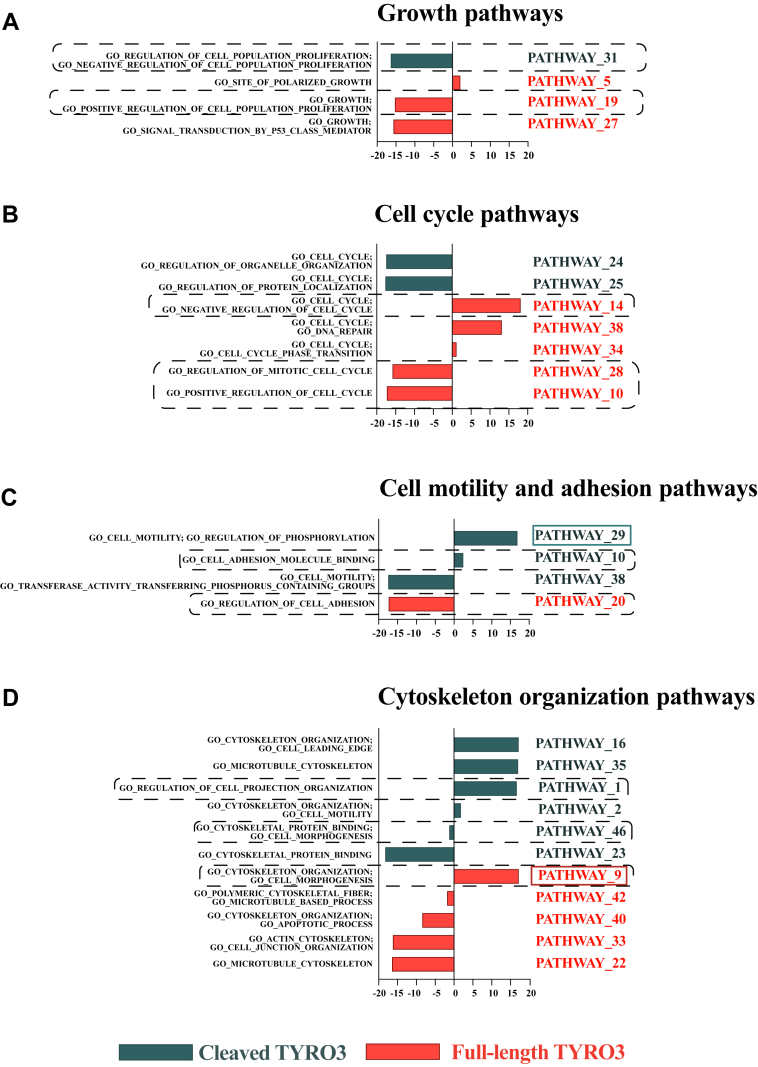
**The functional categorization of the selected full-length and cleaved TYRO3 pathways inferred with the DMPA.** The predicted function of a subset of the multi-omics pathways of the full-length and the cleaved intracellular domain (ICD) of TYRO3 inferred with DMPA. Signaling pathways predicted to regulate the cell growth (*A*), cell cycle (*B*), cell motility and adhesion (*C*) and cytoskeleton organization (*D*) are visualized. The bars represent the median pseudolog2 fold change of the abundance of all the proteins, transcripts, and phosphorylated residues in the pathway against the control condition. For a full list of predicted functions for the pathways of full-length and cleaved ICD of TYRO3, see supplemental Fig. S21. For further details on the pathways, see supplemental Tables S1 and S2. The cell behavior predicted by the pathways surrounded with the *dashed line* is examined in Figure 6, *A*–*C*. The pathways highlighted with solid lines are visualized in Figure 6, *D* and *E*. DMPA, *de novo* multi-omics pathway analysis.

The summary of the predicted functions for each pathway associated with the signaling of full-length and ICD of TYRO3 is displayed in Figure 5 and supplemental Fig. S24 (supplemental Table S10). The functional enrichment analysis of the modeled pathways of full-length and ICD of TYRO3 was able to identify unique pathways associated with various cellular processes including ones involved in the pathogenesis of cancer. A significant difference was noted in the number and direction of pathways related to growth and cell cycle, cell adhesion and motility, cell morphology, cell death, and immune response between the cells producing the full-length *versus* the ICD of TYRO3 (Fig. 5). These findings indicate that a differential response to these functions could be enacted by the full-length and cleaved ICD of TYRO3.

### Functional Validation of the Functional Enrichment Analyses of the DMPA

**Fig. 6:**
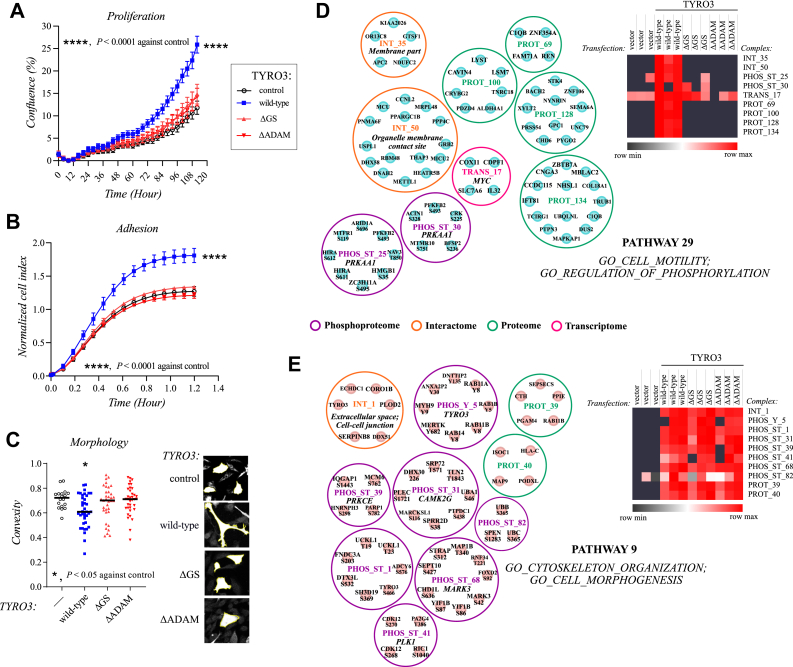
**Functional validation and composition of the selected pathways of full-length and cleaved ICD of TYRO3.***A*, proliferation of WM-266-4 transfectants was measured with live-cell imaging. For statistical testing, the non-parametric Friedman 2-way ANOVA and the Dunn’s multiple comparison test was utilized. The symbols represent the mean and the whiskers the SD of the values. A representative plot from 1 out of 3 independent replicate experiments is shown (n = 6). *B*, real-time adhesion of WM-266-4 transfectants was explored with the xCELLigence cell impedance measurement system. For statistical testing, the parametric 2-way ANOVA and the Dunn’s multiple comparison test was utilized. The symbols represent the mean and the whiskers the SD of the values. A representative plot from 1 out of 4 independent replicate experiments is shown (n = 5). *C*, the morphology of WM-266-4 transfectants was analyzed from thresholded confocal images taken in plane with the plasma membrane with MorphoLibJ plugin of ImageJ. For statistical testing, the non-parametric Kruskal-Wallis ANOVA was utilized. The post hoc analyses were conducted with the Mann-Whitney U test and the resulting *p*-values were corrected with the method of Benjamini, Krieger, and Yekutieli. Convexity: the ratio between the convex perimeter and the real perimeter. One dot represents 1 cell and the horizontal line the median value. Combined results from four independent experiments are shown. *D* and *E*, visualization of the cell motility pathway 29 of cleaved TYRO3 ICD (*D*) and the cytoskeleton organization pathway 9 of full-length TYRO3 (*E*). The median expression of all the transcripts, proteins, or phosphorylated residues in the indicated samples and network modules is presented in the heatmaps. Individual complexes are separated by circles. The inferred network modules are identified by a letter combination (INT for interactome, PHOS_ST for serine/threonine phosphoproteome, PHOS_Y for tyrosine phosphoproteome, TRANS for transcriptome, PROT for proteome) and a number. Predicted transcription factors, kinases, and subcellular locations are indicated with italic letters under the complex identifier. Control: an empty plasmid that does not encode TYRO3 has been transfected to cells. ΔGS: mutation in the γ-secretase cleavage site. ΔADAM: mutation in the predicted ADAM 12/17 cleavage site. ICD, intracellular domain.

To validate the ability of function enrichment of the DMPA to predict differential cellular functions based on molecular data, we randomly selected three cellular functions predicted to be inversly regulated by the signaling pathways associated with the cleaved ICD or the full-length TYRO3. First, to assess proliferation induced by the cleaved ICD or the full-length TYRO3, the growth of WM-266-4 transfectants was analyzed using live-cell imaging (Fig. 6*A*). The function prediction of the ICD pathway 31 and the full-length pathways 19 and 10 (Fig. 5, *A* and *B*) indicated that the cleavage and release of the ICD of TYRO3 would promote cellular growth, while the expression of the full-length TYRO3 would reduce proliferation. Indeed, the WM-266-4 cells expressing WT TYRO3 proliferated significantly faster than the vector control cells or cells expressing the cleavage resistant ΔGS or the ΔADAM variants of TYRO3 that produce less cleaved ICD (Fig. 6*A*).

As another read-out to validate the functional enrichment analyses of the pathways inferred with DMPA, the adhesion rate of the WM-266-4 transfectants to fibronectin-coated wells was investigated by real-time cellular impedance measurement (Fig. 6*B*). As predicted by the ICD pathway 10 and the full-length pathway 20 (Fig. 5*C*), the cells expressing WT TYRO3 adhered to fibronectin with a greater affinity than vector control cells or cells expressing either the ΔGS or ΔADAM variant of TYRO3 (Fig. 6*B*).

Finally, the two-dimensional cellular morphology of the WM-266-4 transfectants was examined from thresholded confocal images with the ImageJ MorphoLibJ plugin (Fig. 6*C*). As indicated by the ICD pathway 46 and the full-length pathway 9 (Fig. 5*D*), the cells expressing WT TYRO3 exhibited distinct morphology from the vector control cells or cells expressing either the ΔGS or ΔADAM variant of TYRO3 (Fig. 6*C*). The morphological difference was captured by the convexity measure (the ratio between the cell area and its convex area), the lower value of which suggests a shape with more protrusions. A greater amount of cell protrusions in the cells expressing WT TYRO3 was also predicted by the DMPA (ICD pathway 1 in Fig. 5*D*). Furthermore, the WM-266-4 cells expressing WT TYRO3 or either of the TYRO3 mutants demonstrated less circular cell shape than the vector control cells, indicating that the full-length TYRO3 additionally regulates cell morphology as suggested by the full-length pathway 9 (supplemental Fig. S25). Taken together, these validation experiments suggest that the function enrichment of the pathways modeled with the DMPA can predict both the function and the direction of the function from purely molecular multi-omics data.

## Discussion

The increased demand for a ground-up multi-omics cell signaling pathway inference method motivated us to create DMPA. The DMPA is based on the calculation and combination of two robust metrics of molecular association, the correlation and stoichiometry score. The inferred molecular associations are consequently modeled into network modules in the DMPA by following the nearest neighbor principle. The DMPA consistently found relevant biological associations in both published and freshly acquired multi-omics data. The DMPA showed consistent performance and the combined score-based networks outperformed correlation-based network models in all tested data types. DMPA outperformed previous methods in both module and pathway discovery.

The advantages of the DMPA include its independence on previous knowledge, robustness, which makes it easily scalable to other omics data types, low computational demand, and ability to work with low sample sizes. DMPA was designed to handle the heterogeneity of multi-omics data and therefore can handle more diverse data types than the previous methods such as WGCNA and GENIE3. Indeed, WGCNA, LOPC, and GENIE3 were unable to make inferences from zero-inflated interactome data. WGCNA, LOPC, and GENIE3 were initially developed to infer gene regulatory modules and networks from gene expression data. DMPA, however, was able to discover reported molecular associations more consistently than WGCNA, LOPC, and GENIE3 from gene expression data and outperformed the other methods in 6 out of 10 datasets. The dimensionality of the omics dataset was a less significant limitation to DMPA, since DMPA was able to infer reported molecular relationships from datasets with small sample sizes (n = 6-30). WGCNA and GENIE3, in contrast, were able to infer molecular relationships only from gene expression datasets of sample sizes 30 or more. Datasets of sample sizes 10 or more, however, are recommended for optimal DMPA performance.

Previous multi-omics integration methods have been focused mostly on feature extraction and matrix factorization strategies. While the inferred latent factors have been found accurate in stratifying samples into clusters and can be predictive of clinical outcome, there is little evidence that the discovered factors would represent signaling pathways in the cell (15, 16). Here we provide evidence that the pathways inferred with the DMPA reflect previously described signaling pathways. DMPA was benchmarked against two previously proposed prior data independent multi-omics integration methods TransNet and multi-omics WGCNA. Of these three methods, DMPA was the only one that was able to discover previously reported cell signaling pathways. This is partly because neither TransNet nor WGCNA have been initially developed for signaling pathway inference, which may indicate that DMPA is the first prior information independent multi-omics integration method designed for this purpose.

Since DMPA is based on the inherent variation between samples in the omics data, the quality of the omics data limits the analysis. High technical variation, significant number of missing values, or low sample size can lead to loss of model accuracy. The nonparametric formulation and a zero-inflated version of the stoichiometry score were devised to partly overcome two of these limitations. Since the correlation, stoichiometry, and combined scores are ranked and thus dependent on the choice of the features included in the dataset, a choice for the combined score cut-off threshold and parameter S and C choice can also significantly affect the final inferred pathways. A script to help the user select optimal C and S parameter values was created to ensure optimized combined score cut-off threshold choice for most datasets. For accurate inference of the multi-omics pathways with DMPA, the multi-omics data should be acquired from the same samples. DMPA does not support combination of multi-omics data that have been acquired from different samples even though they would represent the same tissue type or cell line. DMPA was created to find minimally connecting modules and pathways for all features that have a true association and therefore was not designed to discover all possible true molecular associations in the dataset. Indeed, only the most strongly associated feature connections for most features are likely to be uncovered by the DMPA. Additionally, the DMPA was designed to only uncover linear relationships, and the directionality of the relationships is similarly not currently solved by the DMPA.

Here a new association measure, the stoichiometry score was introduced. While the stoichiometry score overall did have poorer performance than the combined score, the stoichiometry score showed promise as an association measure for mass spectrometry–derived interactome data. In some datasets the stoichiometry score was able to discover more reported molecular associations of high confidence than the combined score indicating that there may be benefits to solely using the stoichiometry score as an association measure for certain types of interactome datasets. More research, however, is needed to define these datasets. Combining the stoichiometry score with correlation produced a more consistent molecular association measure, the combined score. Here, evidence was provided that the combined score indeed can predict molecular association in omics datasets.

We acquired multi-omics data from the cleavage-resistant and WT TYRO3 variants and utilized the DMPA to shed light on the unknown cleavage-associated signaling pathways of TYRO3 in melanoma cells. In addition to signaling as a full-length receptor, TYRO3 has been proposed to undergo regulated intramembranous proteolysis by sequential proteolysis by the gamma-secretase complex followed by cleavage by an ADAM protease, releasing a soluble ICD with potential signaling activities (67). The DMPA discovered a total of 51 and 46 differential unique signaling pathways for the full-length TYRO3 and the cleaved TYRO3 ICD, respectively. An enrichment analysis to infer the biological process the pathways were involved with was also devised. A difference in the number and the direction of the pathways associated with growth and cell cycle, cell motility and adhesion, immune responses, chromosome organization, cell death, and cell differentiation were observed between the full-length and cleaved TYRO3 ICD. Many of these functions may be critically involved, for example, in the progression or therapeutic responses in melanoma. For example, TYRO3 has been implicated in the proliferation, tumorigenesis, chemoresistance, and motility of melanoma cells (71, 76, 77, 78). Validation experiments confirmed that the DMPA with the function prediction was able to accurately predict both the function and the direction of the function of the cells expressing either WT or cleavage-resistant variants of TYRO3. These findings also represent the first comprehensive characterization of signaling pathways stimulated by the gamma-secretase–released ICD of a receptor tyrosine kinase as compared to the canonical signaling *via* the full-length receptor.

The DMPA segregates all signaling events into pathways independent on the amount of previous knowledge on the feature. This aspect is crucial in the discovery of novel signaling modules. Since DMPA can predict molecular associations, it can predict upstream transcription factors of transcripts, kinases of phosphorylation sites, and subcellular locations of proteins in the modules, for which these regulators and subcellular locations are unknown. Similarly, the DMPA can predict connections to biological functions for proteins, transcripts, or post-translationally modified proteins in the pathways for which no previous connection to the biological function has been discovered. The pathways inferred with DMPA can be re-entered into DMPA to acquire super pathways and thus broader connections between the modeled pathways can additionally be discovered with the DMPA.

## Data Availability

The mass spectrometry–derived proteomics data have been deposited to the ProteomeXchange Consortium *via* the PRIDE partner repository with the dataset identifier PXD046503. The RNAseq data have been deposited to the Gene Expression Omnibus database with the identifier: GSE190431. The DMPA is available both as matlab code and executables in Mendeley Data: https://data.mendeley.com/datasets/m3zggn6xx9/draft?a=71c29dac-714e-497e-8109-5c324ac43ac3, (https://doi.org/10.17632/m3zggn6xx9.1). The source data for Figure 6, *A* and *B* is available as supplemental Table S13 that includes the numerical values of all replicate experiments.

## Supplemental Data

This article contains supplemental data (33, 34, 35, 36, 37).

## Conflict of interest

The authors declare that they have no conflicts of interest with the contents of this article.
