# Supplementary material for: De Novo Multi-Omics Pathway Analysis Designed for Prior Data Independent Inference of Cell Signaling Pathways
Source: Mol Cell Proteomics. 2024 May 3;23(7):100780. doi: 10.1016/j.mcpro.2024.100780 (PMC11259815; doi:10.1016/j.mcpro.2024.100780)

De Novo multi-omics pathway analysis (DMPA) designed for prior data independent inference of cell signaling pathways

Katri Vaparanta[1, 2, 3], Johannes A. M. Merilahti[1, 2, 3], Veera K. Ojala [1, 2, 3], and Klaus Elenius[1,2,3,4].

[1] *Turku Bioscience Centre, University of Turku and Åbo Akademi University, 20520 Turku, Finland*

[2] *Medicity Research Laboratories, University of Turku, 20520 Turku, Finland*

[3] *Institute of Biomedicine, University of Turku, 20520 Turku, Finland*

[4]*Department of Oncology, Turku University Hospital, Turku, 20521, Finland*

List of the included Supplemental Data:

S-5: Figure S1. The parameters of the network module and pathway inference of DMPA.

S-6: Figure S2. Additional validation of interactome network modules inferred with DMPA.

S-7: Figure S3. Additional validation of transcriptome network modules inferred with DMPA.

S-8: Figure S4. Validation of the network modules inferred with DMPA with additional omics data.

S-9: Figure S5. Validation of the design decisions of DMPA.

S-10: Figure S6. Effect of the size parameters 7 and 9 on the inferred network modules and performance of DMPA.

S-11: Figure S7. Effect of parameter S choice on the performance of DMPA with transcriptome data.

S-12: Figure S8. Effect of parameter S choice on the performance of DMPA with normally distributed simulated data.

S-13: Figure S9. Effect of parameter S choice on the performance of DMPA with negative binomially distributed simulated data.

S-14: Figure S10. Effect of parameter S choice on the performance of DMPA with beta distributed simulated data.

S-15: Figure S11. The determinants of accurate S parameter value choice.

S-16: Figure S12. Sensitivity analyses with different C parameter values.

S-17: Figure S13. The effect of the zero-inflated version of DMPA on the performance of DMPA with zero-inflated and non-zero-inflated data.

S-18: Figure S14. The effect of sample size and feature set size on the performance of DMPA.

S-19: Figure S15. Functional validation of cleavage-resistant TYRO3 receptor variants.

S-20: Figure S16. Interactome of full-length TYRO3 and cleaved TYRO3 ICD.

S-21: Figure S17. Differentially expressed phosphoproteome of full-length TYRO3.

S-22: Figure S18. Differentially expressed phosphoproteome of cleaved TYRO3 ICD.

S-23: Figure S19. Differentially expressed transcriptome of full-length TYRO3 and cleaved TYRO3 ICD.

S-24: Figure S20. Differentially expressed proteome of full-length TYRO3.

S-25: Figure S21. Differentially expressed proteome of cleaved TYRO3 ICD.

S-26: Figure S22. Network modules of full-length TYRO3.

S-27: Figure S23. Network modules of cleaved TYRO3 ICD.

S-28: Figure S24. The functional categorization of the full-length and cleaved TYRO3 ICD pathways inferred with DMPA.

S-29: Figure S25. Morphology of WM-266-4 transfectants.

S-30–33: Pseudocode of DMPA

S-34: Uncropped western blots of Figure S15

Table S1 (individual Excel file): The signaling pathways of full-length and cleaved TYRO3 inferred with DMPA.

Table S2 (individual Excel file): The network modules of full-length and cleaved TYRO3 inferred with DMPA.

Table S3 (individual Excel file): The predicted transcription factors for the transcription modules of full-length and cleaved TYRO3 inferred with DMPA.

Table S4 (individual Excel file): The predicted subcellular locations of the interactome modules of full-length and cleaved TYRO3 inferred with DMPA.

Table S5 (individual Excel file): The predicted upstream kinases of the phosphoproteome modules of full-length and cleaved TYRO3 inferred with DMPA.

Table S6 (individual Excel file): The normalized LFQ intensity values for the proteins that differentially co-precipitated with the wild-type and cleavage-resistant variants of TYRO3.

Table S7 (individual Excel file): The normalized LFQ intensity values for the differentially phosphorylated residues in the cells expressing wild-type and cleavage-resistant variants of TYRO3.

Table S8 (individual Excel file): The transcripts per million values for the transcripts that were differentially expressed in cells expressing the wild-type and cleavage-resistant variants of TYRO3.

Table S9 (individual Excel file): The normalized LFQ intensity values for the proteins that were differentially expressed in cells expressing the wild-type and cleavage-resistant variants of TYRO3.

Table S10 (individual Excel file): The predicted functions of the signaling pathways of full-length and cleaved TYRO3 inferred with DMPA.

Table S11 (individual Excel file): The expression values of the network modules of full-length and cleaved TYRO3 inferred with DMPA.

Table S12 (individual Excel file): The proteins, phosphorylated proteins and phosphorylated peptides identified in the MS/MS.

Table S13 (individual Excel file): Raw numerical data for the growth and adhesion experiments in Figure 6A-B.

Table S14 (individual Excel file): Description of the datasets used for DMPA validation.

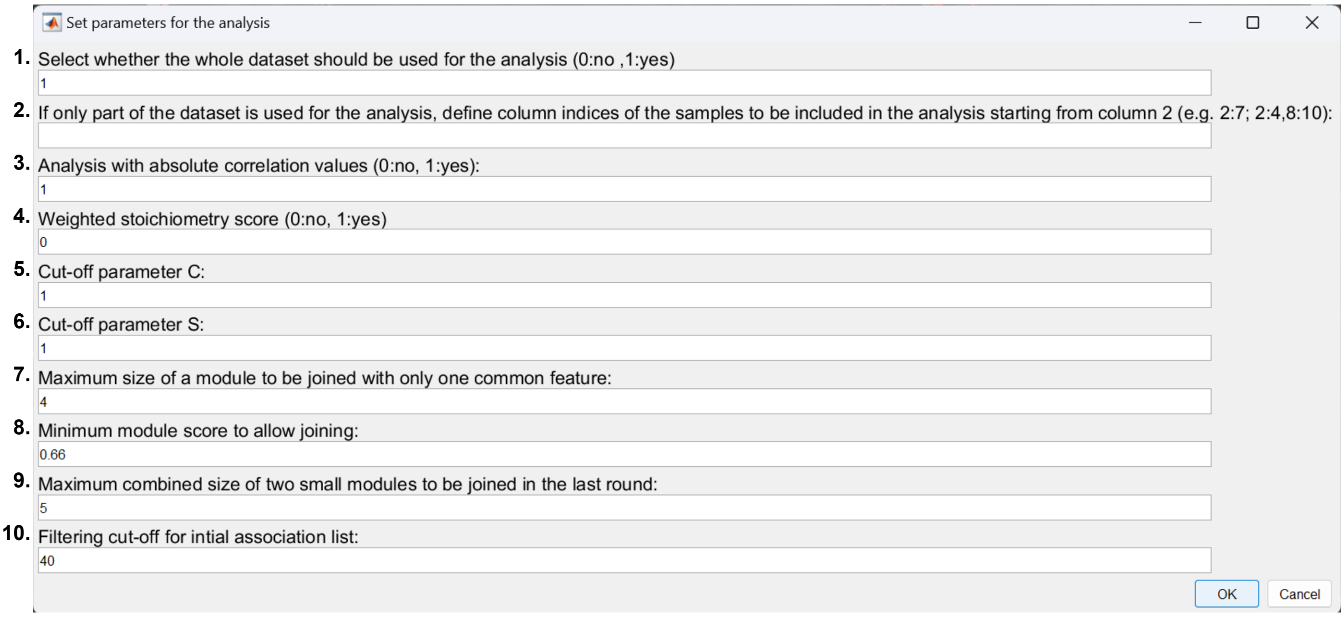

Figure S1. The parameters of the network module and pathway inference of DMPA. 1-2: The dataset is defined for the inference. **3:** The use of absolute values of the unadjusted correlation score is defined. The absolute values should be used if both positive and negative relationships are of interest. **4:** The use of weighted or non-weighted unadjusted stoichiometry score is defined. Weighted stoichiometry score should be used with zero-inflated data. **5:** The value for the cut-off parameter C is defined. The cut-off parameter C controls the cut-off threshold for the combined score by defining the minimum number of feature pairs for one feature that should be considered in the inference. The cut-off parameter C should be set based on the recommendations of the suggest parameters for module and pathway inference function. **6:** The value for the cut-off parameter S is defined. The cut-off parameter S controls the cut-off threshold for the combined score by defining the number of features for which no association need to be considered in the analysis. The parameter S value should reflect the expected number of features with no true association in the dataset. The cut-off parameter S should be set based on the recommendations of the suggest parameters for module and pathway inference function. **7:** The maximum size of a module for joining based on only one common feature is defined. Adjusting this parameter will increase or reduce the size of the final modules. Can be freely left to default value. Can also be set to 0 if no joining based on only one common feature is desired. **8:** Score threshold for module joining based on only one common feature is defined. This filtering step ensures that modules with low scores are not joined with high scoring modules. Can be freely left to the default value or adjusted if more stringent rules for joining are desired. **9:** The maximum combined size for last round of module joining based on only one common feature is defned. This step is redundant if joining controlled by the parameter 7 is not allowed. Can be freely left to default value. Can also set to 0 if no joining based on only one common feature is desired. **10:** The maximum allowed score difference for filtering based on combined score discrepancy is defined. This parameter controls the maximum allowed combined score difference for feature pairs. Feature pairs for which the adjusted combined score of feature A for feature B is significantly higher or lower than the adjusted combined score of feature B for feature A are filtered out from the inference. The score difference is defined by the expected score difference of x number of features. The x is the value that needs to be supplied. The default value 40 corresponds to a score difference of 0.4 for feature set size of 100.

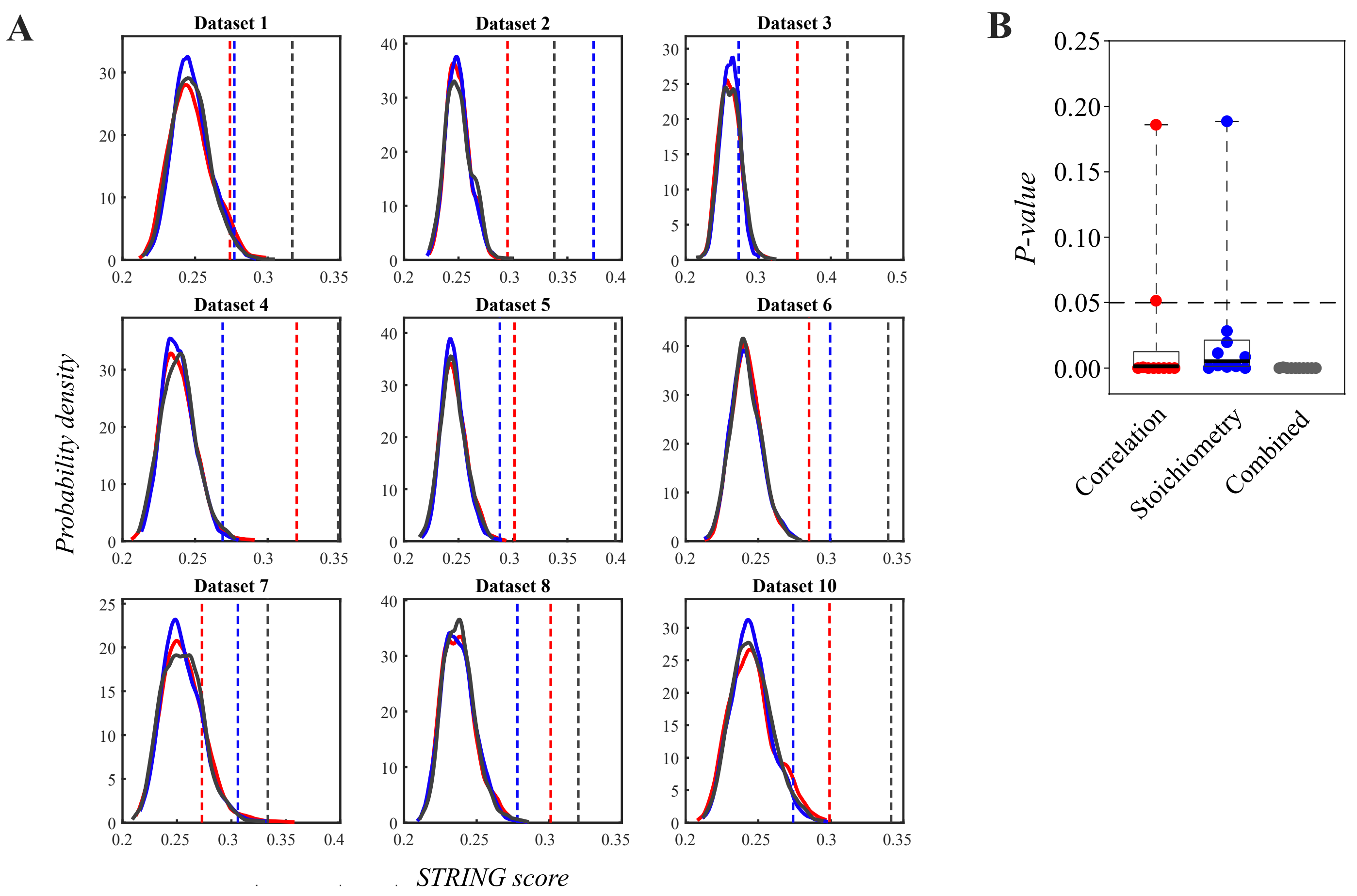

Figure S2. Additional validation of interactome network modules inferred with DMPA. Empirical probability densities (**A**) simulated by randomizing the proteins of the inferred interactome network modules into modules of the same size and the corresponding P-values (**B**) for the validation scores of the network modules inferred with DMPA. The modules were inferred from interactome data by utilizing either the correlation (Corr, red), the stoichiometry (Stoi, blue) or the combined score (Comb, grey). The median values of the STRING score for the protein-protein interactions inside the modules as determined by the STRING protein-protein interaction database were used to determine the empirical probability densities and the validation scores for the inferred modules. The empirical probability densities are visualized with color-coded solid lines and the validation scores for the inferred modules in color-coded dashed lines. One dot in the boxplot represents a P-value for one dataset, the box the interquartile range and the line the median value. The datasets were acquired from published interactome data.

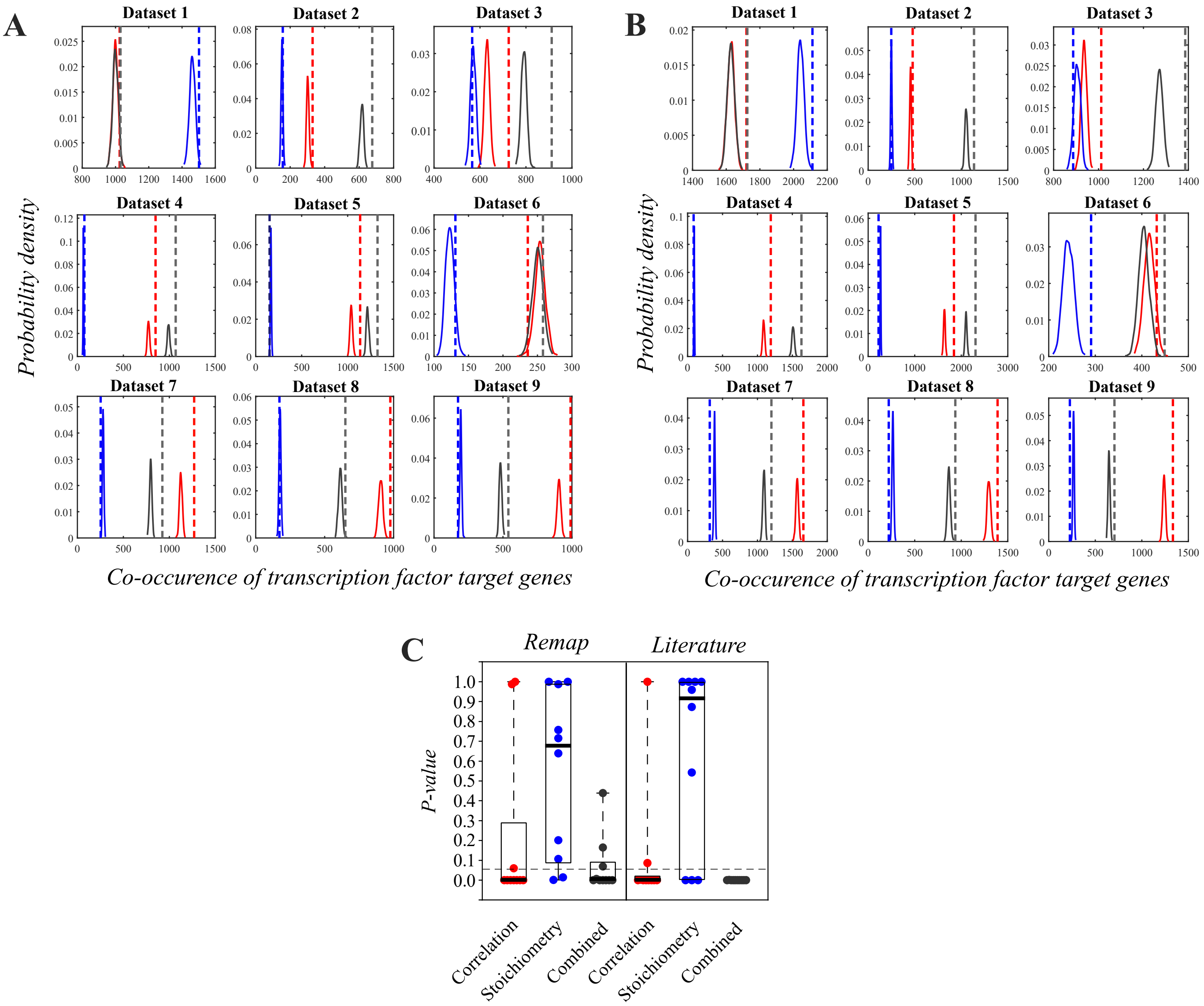

Figure S3. Additional validation of transcriptome network modules inferred with DMPA. Empirical probability densities (**A, B**) simulated by randomizing the transcripts of the inferred transcriptome network modules into modules of the same size and the corresponding P-values (**C**) for the validation scores of the modules inferred with DMPA. The modules were inferred from transcriptome data by utilizing either the correlation (Corr, red), the stoichiometry (Stoi, blue) or the combined score (Comb, grey). The sum of co-occurrences of transcripts reported to be regulated by the same transcription factor in the modules were used to determine the empirical probability density and the validation score for the inferred modules. The transcription factor target gene relationships were acquired from Remap (**A**) and Literature (**B**) annotation sets of the ChEA3 database. The empirical probability densities are visualized with color-coded solid lines and the validation score for the inferred network modules in color-coded dashed lines. One dot in the boxplot represents a P-value for one dataset, the box the interquartile range and the horizontal line the median value. The datasets were acquired from

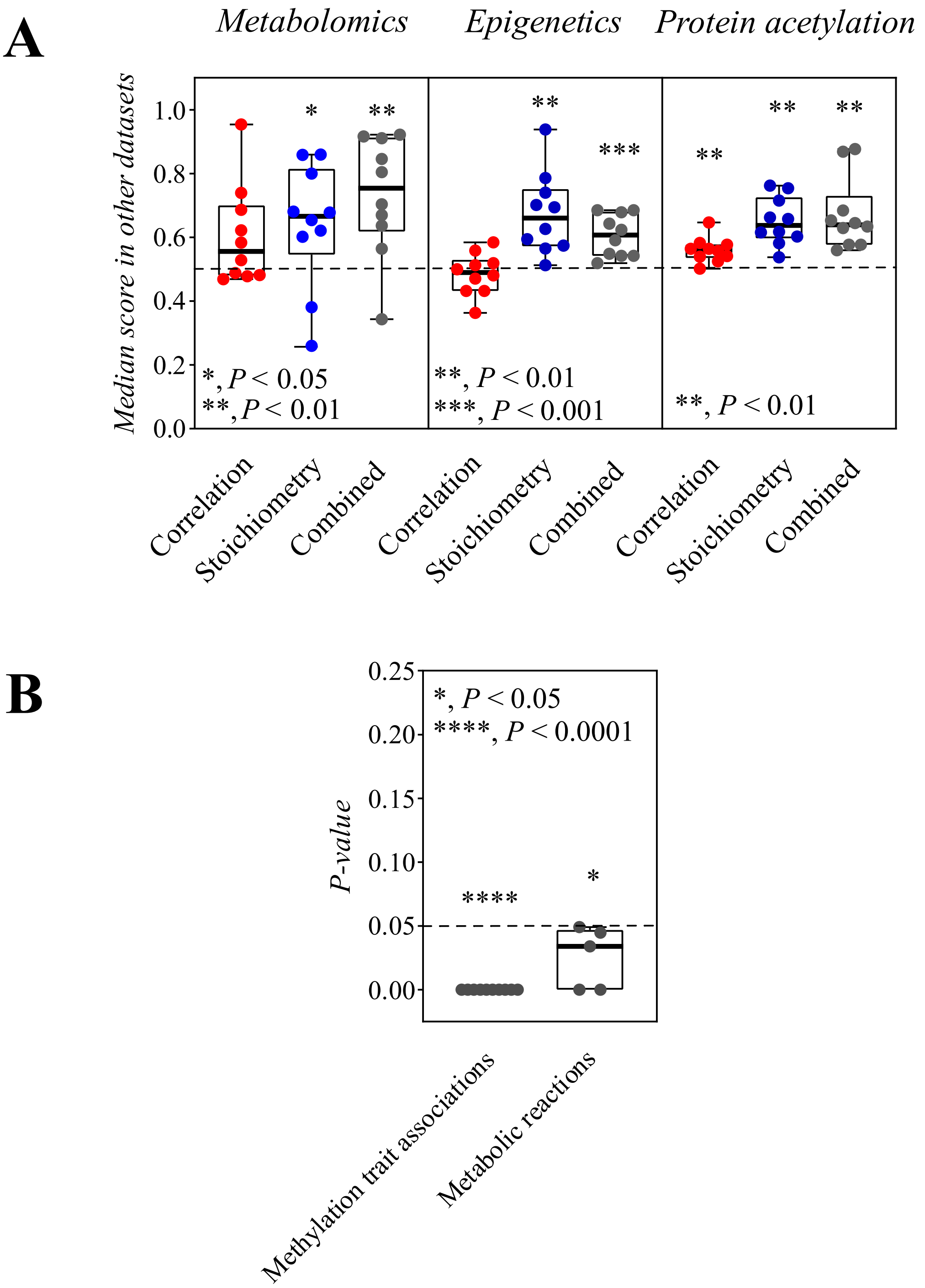

Figure S4. Validation of the network modules inferred with DMPA with additional omics data.
A: Conservation of the indicated scores in metabolomics, epigenome and protein acetylation data. Two-tailed one-sample T-test.
B: Ability of the network modules inferred with DMPA to predict methylation trait associations and metabolic reactions in epigenetics and metabolomics data, respectively. The combined score was used. One-tailed one sample Wilcoxon rank test.
The metabolomics data was acquired from the Metabololights database. The methlyation and protein acetylation data was acquired from the CPTAC data accessed through the LinkedOmics database.

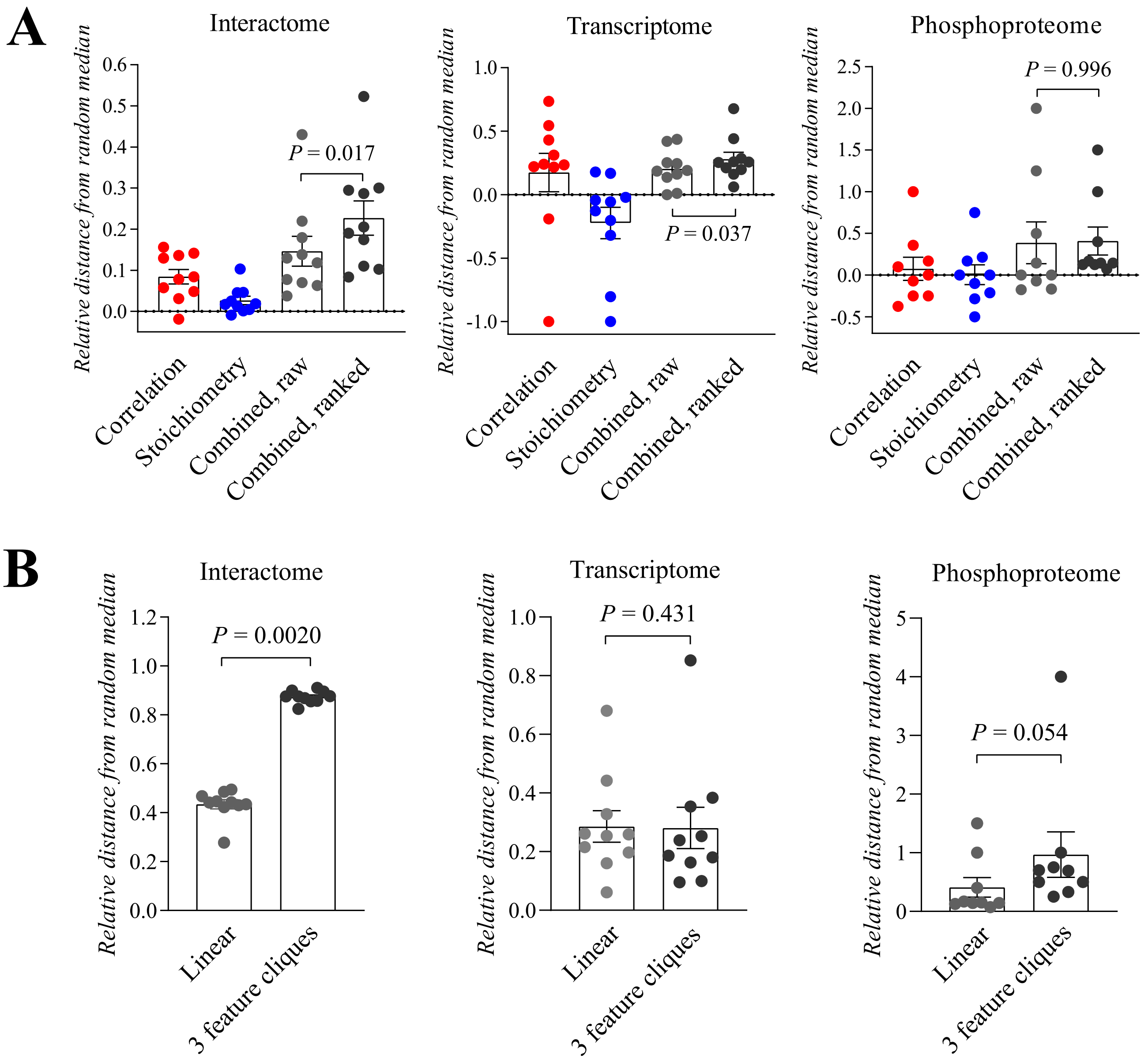

Figure S5. Validation of the design decisions of DMPA.
A: The effect of the stoichiometry score and the correlation score adjustment on the accuracy of DMPA to discover published molecular associations in interactome, transcriptome and phosphoproteome data was examined. The correlation, stoichiometry, unadjusted (raw) combined and ranked combined score were determined for each feature pair and the feature pair with the maximum score for each feature was determined. The frequency of the feature pairs with the maximum score in a set of reported protein-protein interactions, transcripts regulated by the same transcription factor or substrates regulated by the same kinase, as determined by the STRING, ENCODE and PhosphoSitePlus databases, was assessed. Randomized empiricial probability densities were estimated by estimating the same frequency for random pairs. The distance of the frequency from the median of the random density was estimated in each condition. The distance was normalized against the median of the random density. Repeated measures one-way ANOVA with Dunnett's multiple comparisons test.
B: The effect of the 3 feature clique approach on the accuracy of DMPA to discover reported molecular associations in interactome, transcriptome and phosphoproteome data was examined. The 3 feature clique approach was compared to a linear approach where one maximally scoring pair was assigned to each feature. Relative distance from random median was estimated as in A.Wilcoxon matched-pairs signed rank test.

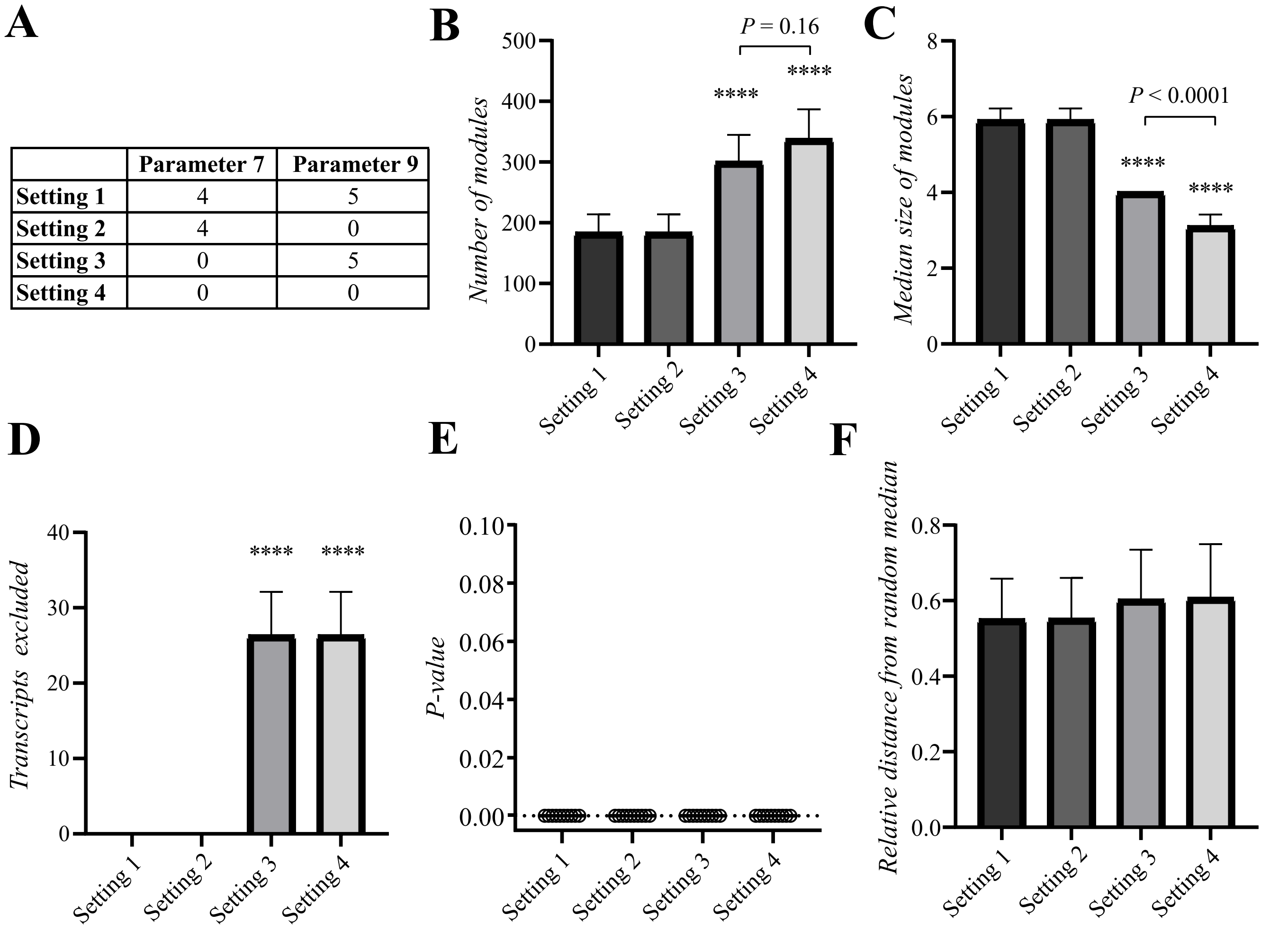

	Parameter 7	Parameter 9
**Setting 1**	4	5
**Setting 2**	4	0
**Setting 3**	0	5
**Setting 4**	0	0

Figure S6. Effect of the size parameters 7 and 9 on the inferred network modules and performance of DMPA. Published transcriptome data was analyzed with DMPA with different parameter 7 and 9 settings. The number and size of the modules and the ability of DMPA to discover reported transcription factor target gene relationships was examined.

A: Parameter values for the size parameters 7 and 9 (Figure S1) in network module inference in different settings.

B: Number of modules with different settings.

C: Median size of network modules with different settings.

D: Number of transcripts excluded from the modules inferred with different settings.

E-F: P-value and the relative distance from the median of the randomized probability distribution of the modules inferred with different settings. The P-value and the relative distance from random median was assessed by quantifying the reported transcription factor target gene relationships as determined by the ENCODE database in the inferred and randomized modules. ****, $P < 0.0001$ against Setting 1. One-way ANOVA and Tukey's multicomparison test was used for statistics. Mean ± SD (n=10).

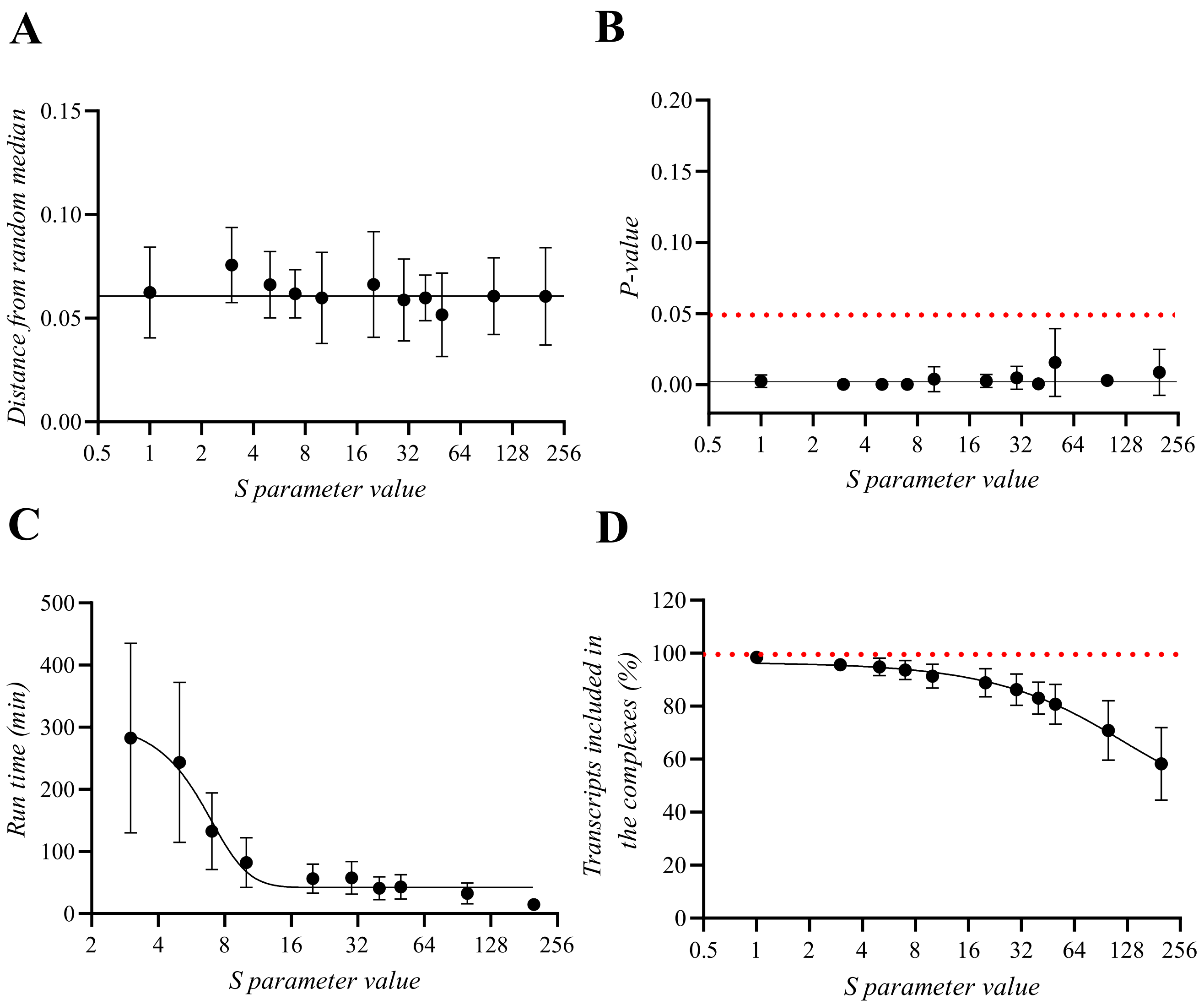

Figure S7. Effect of parameter S choice on the performance of DMPA with transcriptome data.
A-B: The effect of parameter S choice on the on the accuracy of DMPA to discover reported molecular associations in transcriptome data. The relative distance from the randomized median (A) and the corresponding P-value (B) of the validation score (sum of co-occurences of reported molecular associations in the inferred modules) is shown. To estimate the P-value and the relative distance from random median a probability distribution was estimated form the sum of co-occurences of reported associations in randomized modules of the same size.
C-D: The effect of parameter S choice on the runtime (C) of DMPA and number of transcripts included in the modules inferred with DMPA (D).
Mean ± SEM (n=3).

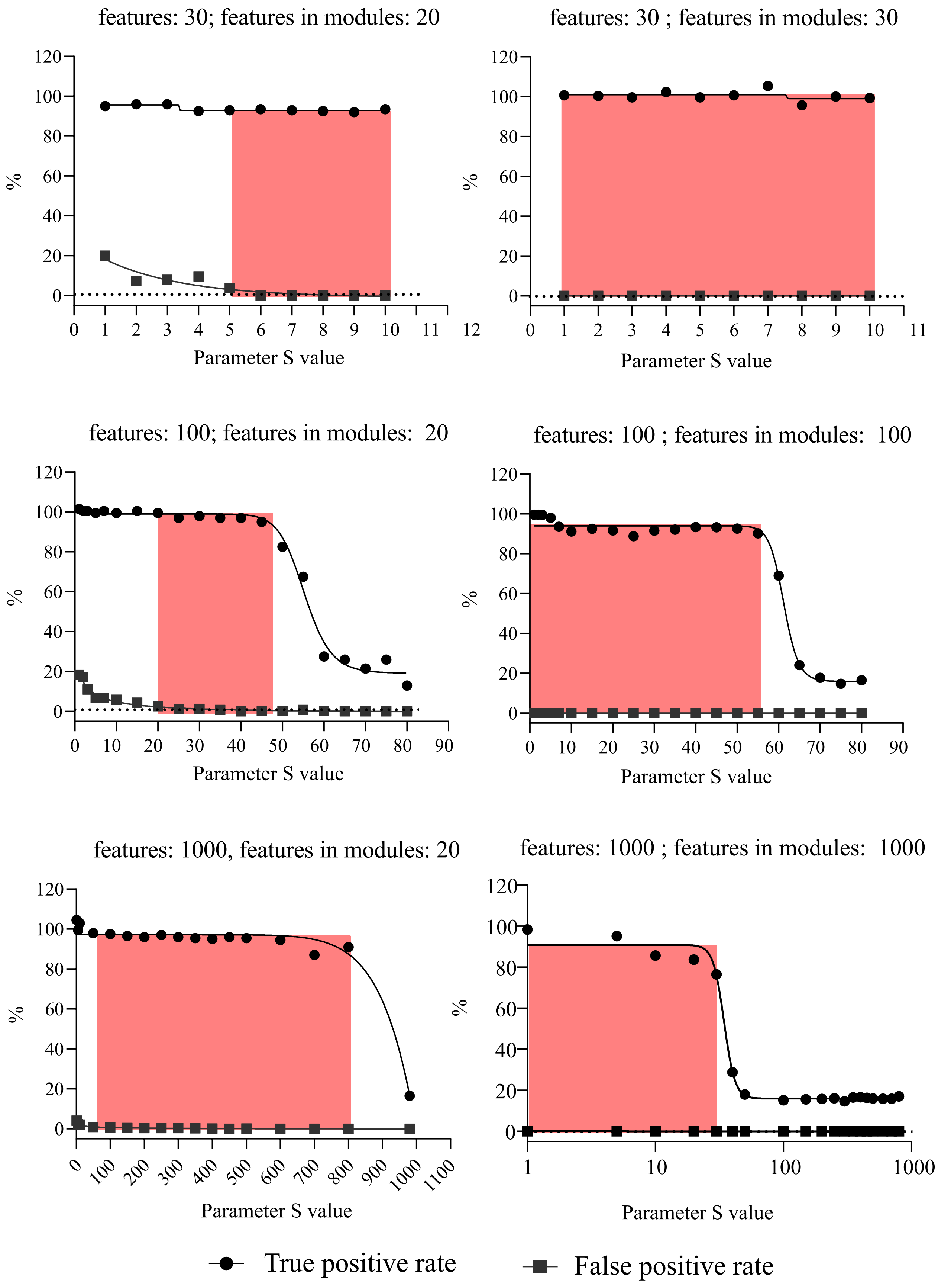

Figure S8. Effect of parameter S choice on the performance of DMPA with normally distributed simulated data. The true and false postive rates at different parameter S values in datasets of different sizes and varying number of features in modules. The optimal value range for the parameter S is indicated with red.

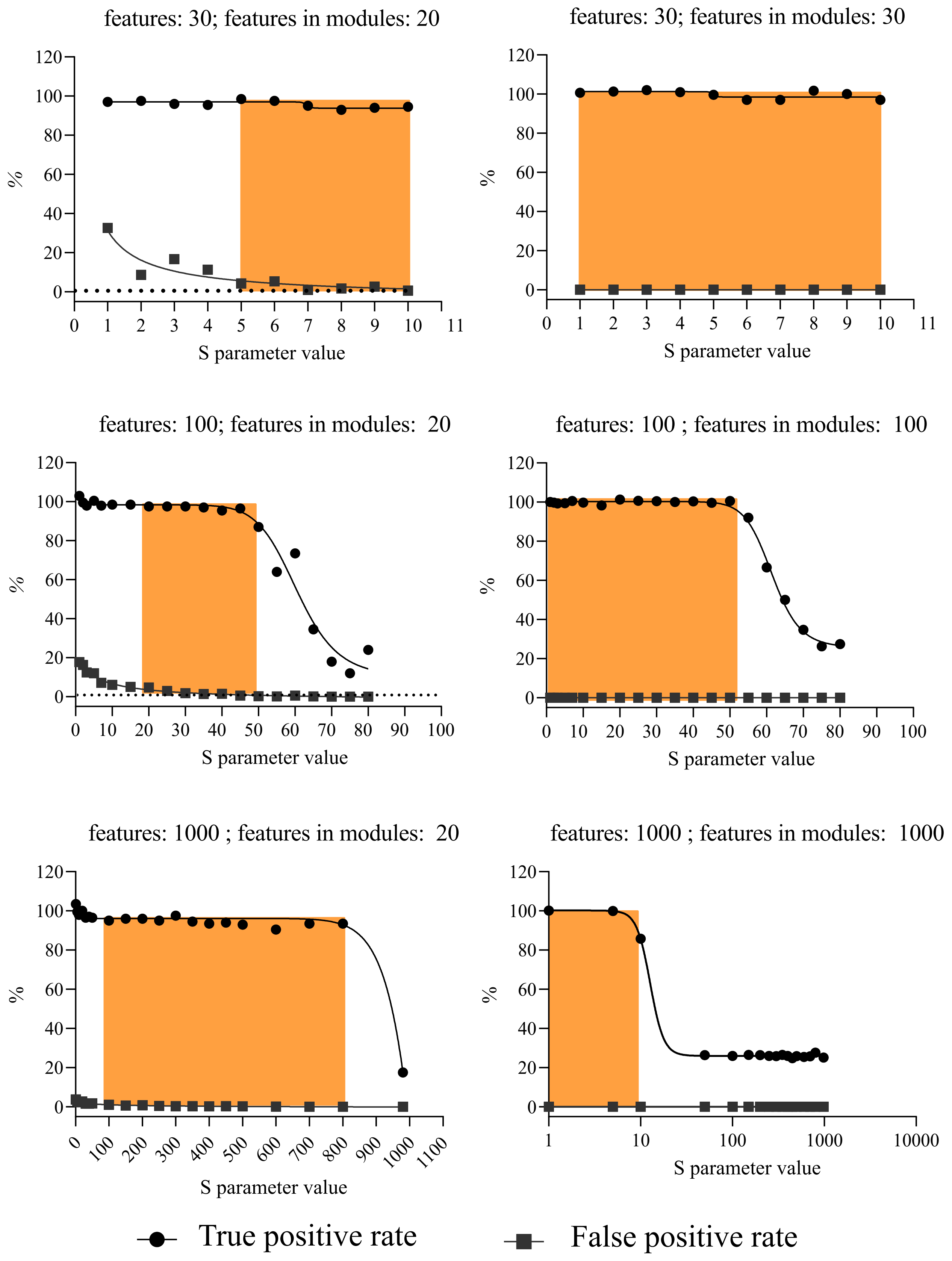

Figure S9. Effect of parameter S choice on the performance of DMPA with negative binomially distributed simulated data. The true and false postive rates at different parameter S values in datasets of different sizes and varying number of features in modules. The optimal value range for the parameter S is indicated with orange.

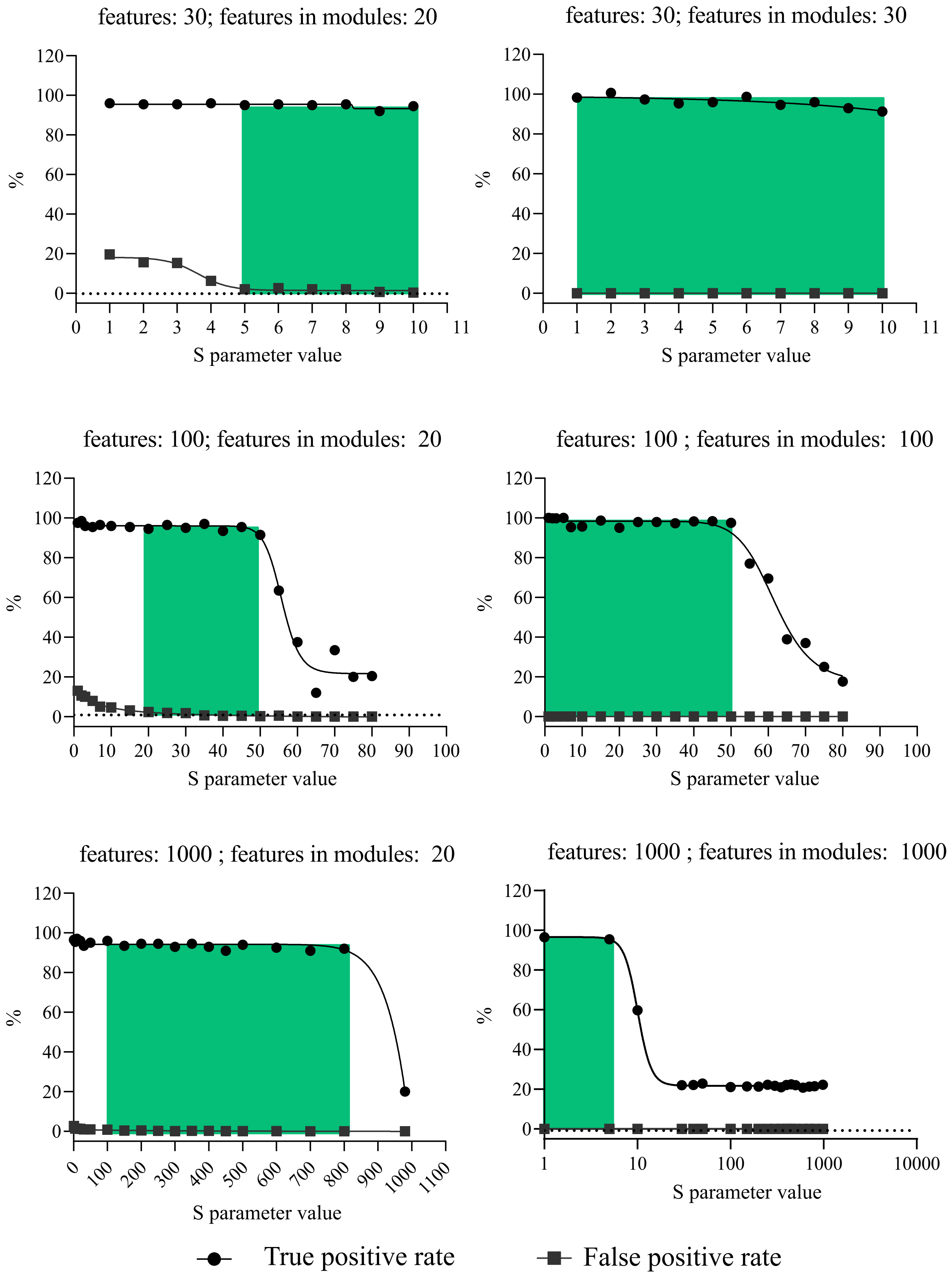

Figure S10. Effect of parameter S choice on the performance of DMPA with beta distributed simulated data. The true and false postive rates at different parameter S values in datasets of different sizes and varying number of features in modules. The optimal value range for the parameter S is indicated with green.

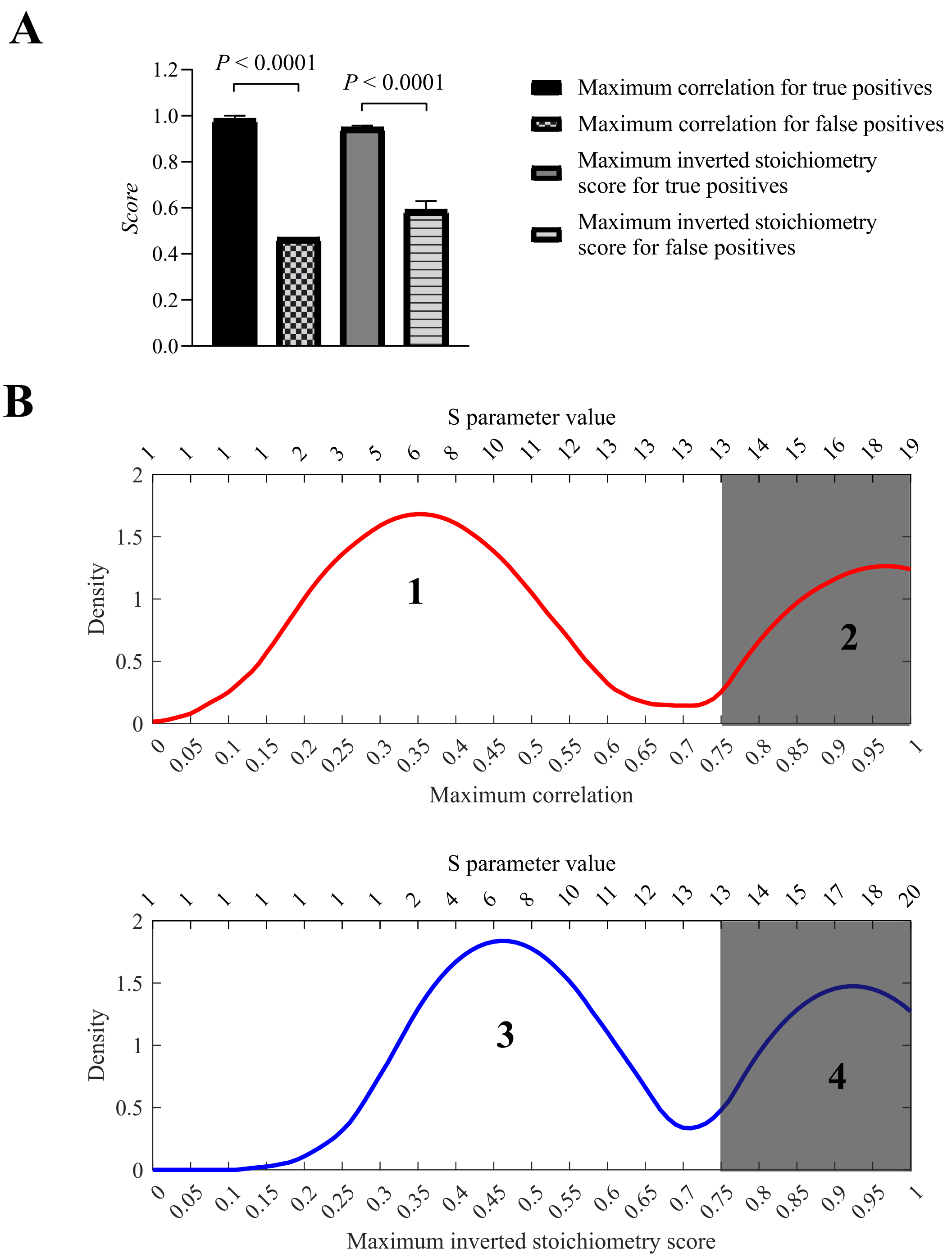

Figure S11. The determinants of accurate S parameter value choice.
A: The median maximum correlation and inverted stoichiometry score for false and true positives in simulated data. Mean ± SD (n=30). Two-tailed Mann-Whitney U-test.
B: The output of the script designed to inform the S parameter value choice. Peak 1 and 3 represent the peaks for the false positives and peaks 2 and 4 the true positives in maximum correlation and inverted stoichiometry score, respectively. The grey area represent the simulated features included in the analysis after cut-off at optimal parameter S value 13 is set for the algorithm for the simulated data. 20 features in modules and 25 features with no true module assignation were simulated.

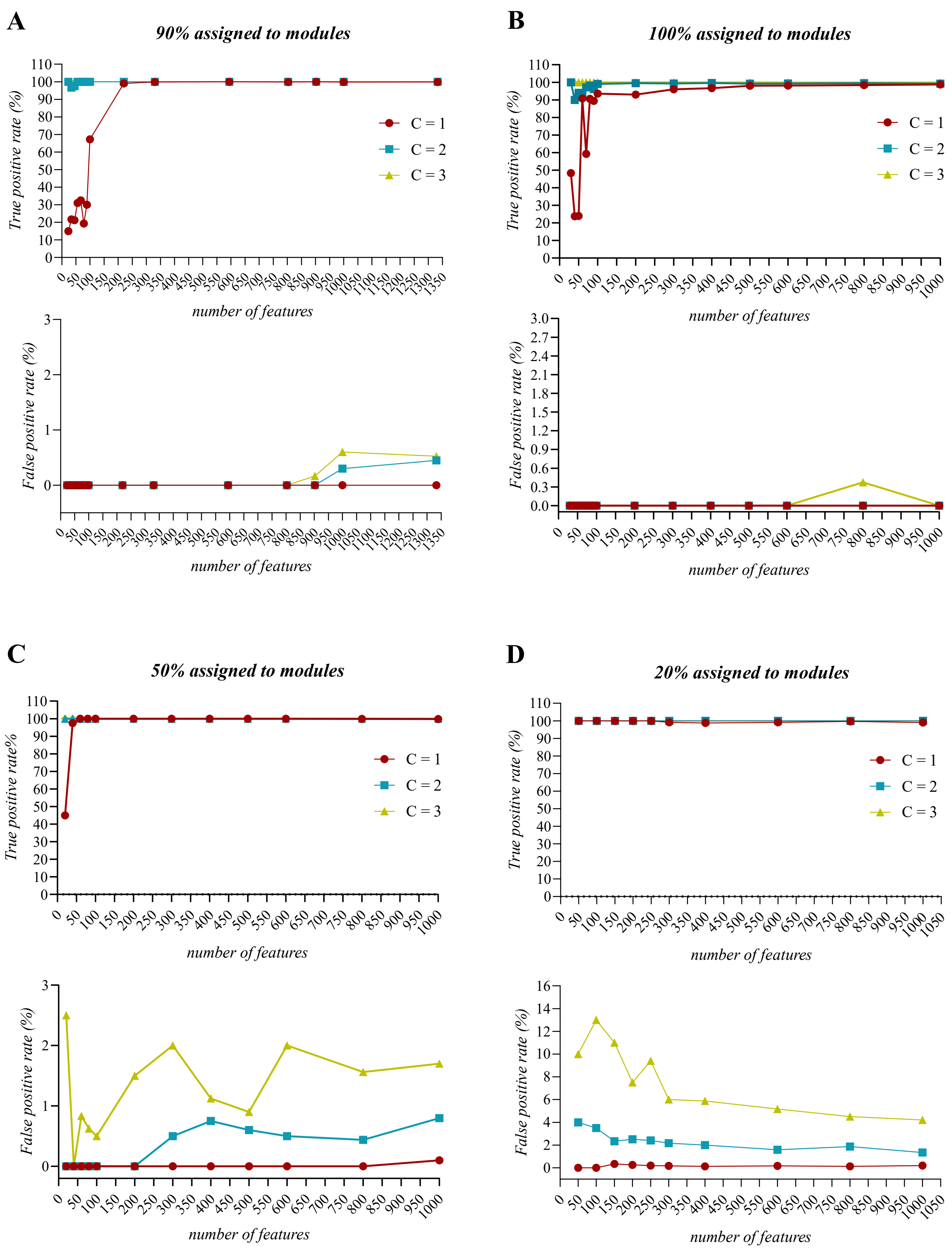

Figure S12. Sensitivity analyses with different C parameter values. The sensitivity of DMPA to parameter C value was examined with simulated datasets. Datasets with different feature set sizes and proportions of non-associated features were subjected to DMPA. The true and false positive rate was examined.

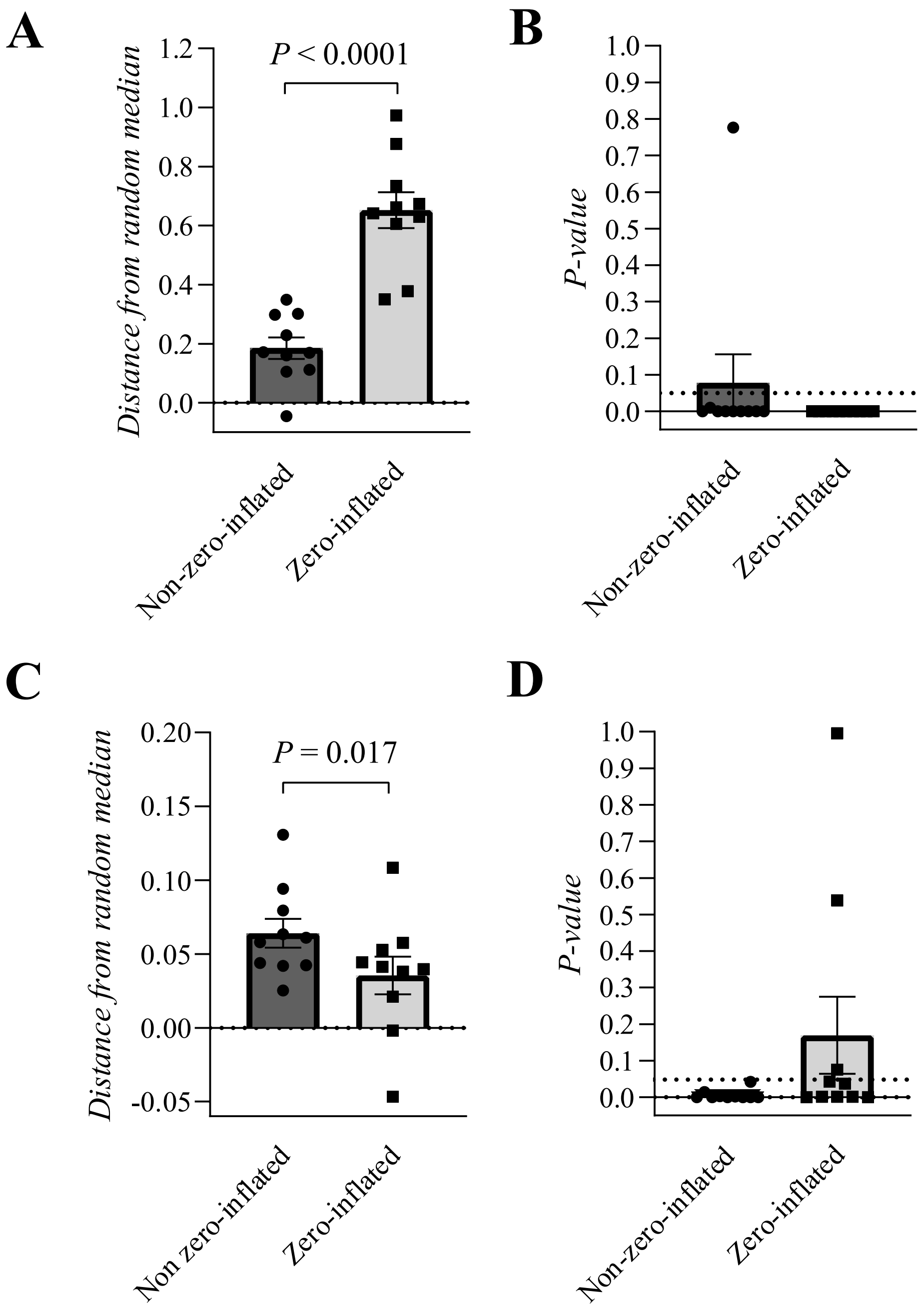

Figure S13. The effect of the zero-inflated version of DMPA on the performance of DMPA with zero-inflated and non-zero-inflated data.

A-B: The effect of the zero-inflated version on the performance of DMPA to discover reported protein-protein interactions in zero-inflated interactome data.

C-D: The effect of the zero-inflated version on the performance of DMPA to discover reported transcription factor target gene relationships in non-zero-inflated transcriptome data.

The relative distance of the sum of co-occurences of reported molecular associations in the inferred modules from the randomized median and the corresponding P-value is shown. To estimate the P-value and the relative distance from random median a probability distribution was estimated form the sum of co-occurences of reported associations in randomized modules of the same size.

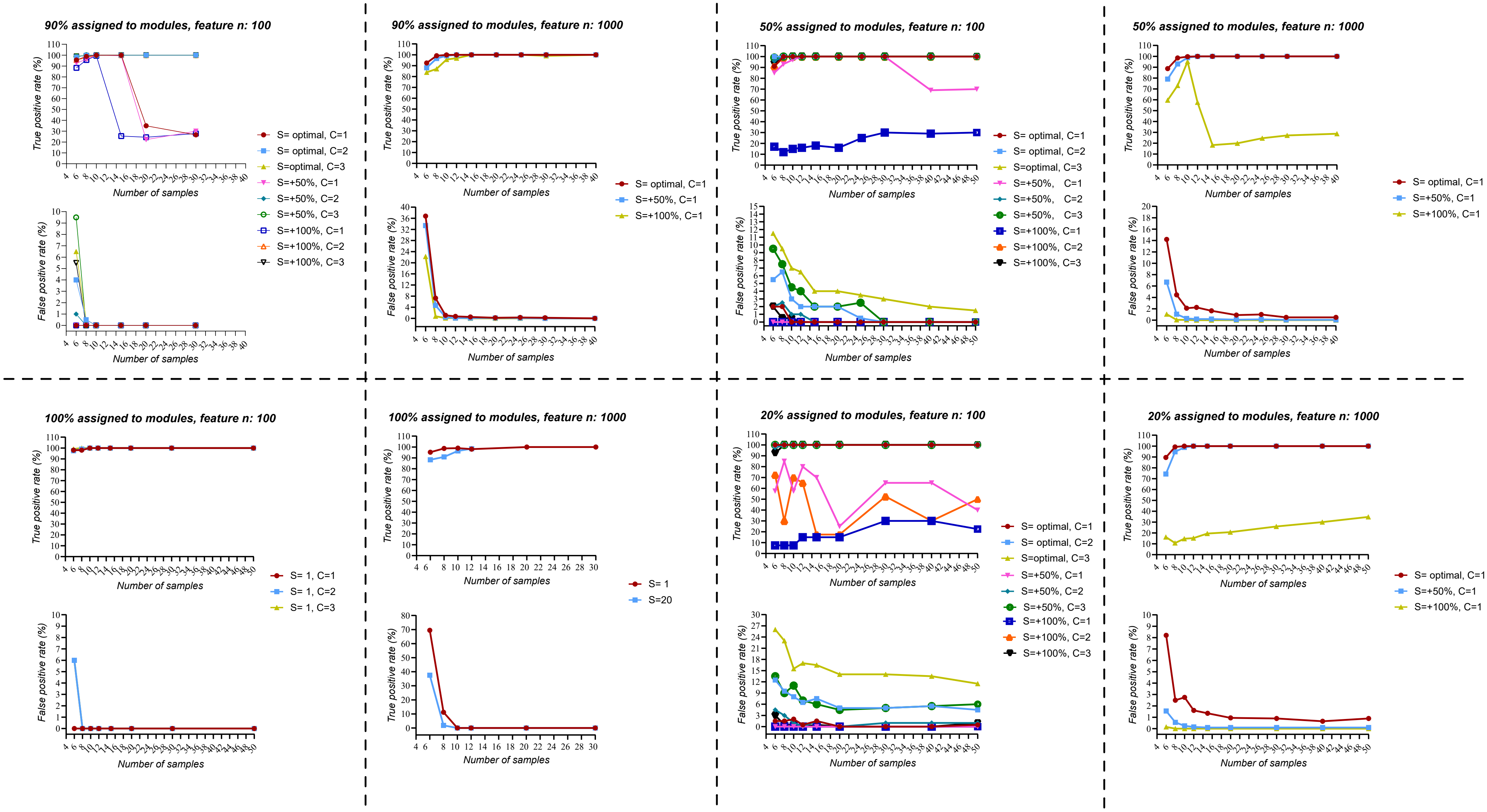

Figure S14. **The effect of sample size and feature set size on the performance of DMPA.** Datasets with variable sample sizes, feature set sizes and percentage of features with no true module assignment were simulated and analyzed with DMPA with different parameter C and S values. The true postive and false positive rates were determined. S=optimal: S has been set to half of the number of features with no true modules assignment in the dataset. S=+50%: S has been set to 50% higher than the optimal value. S=+100%: S has been set to 100% higher than the optimal value.

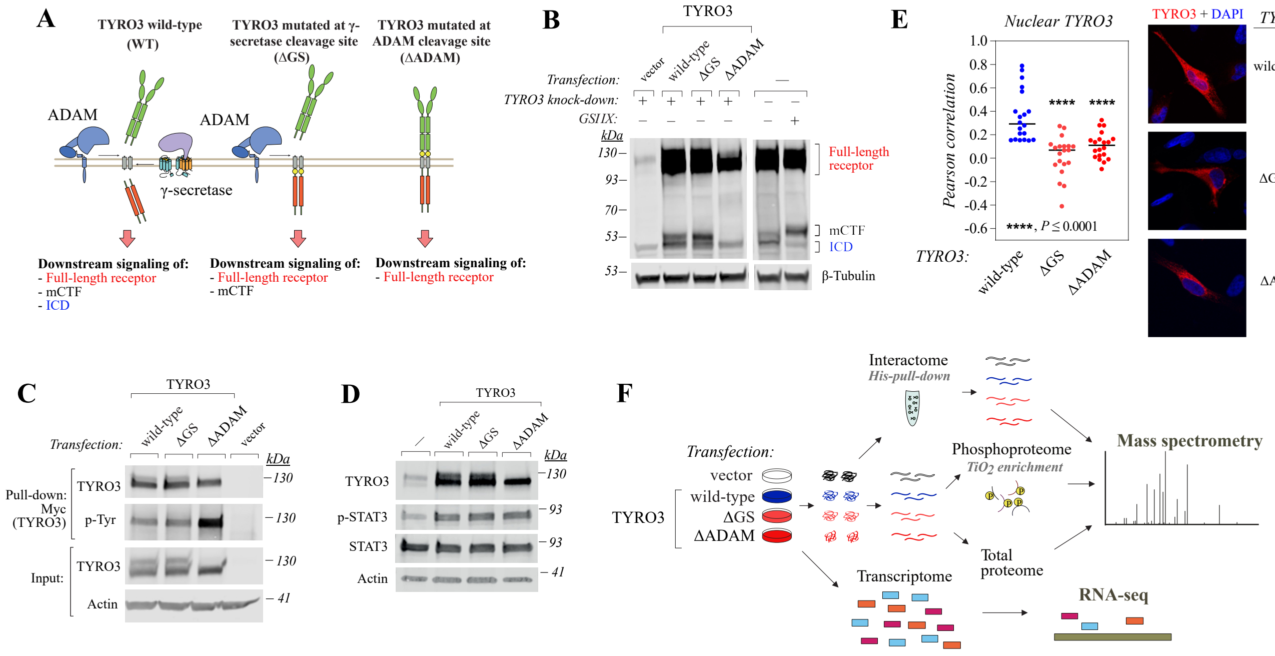

Figure S15. Functional validation of cleavage-resistant TYRO3 receptor variants. A: A Schematic portraying the signaling mediated by wild-type and regulated intramembrane proteolysis (RIP)-resistant variants (ΔGS, ΔADAM) of TYRO3. Since the cleavage events in RIP are sequential, blocking only the gamma-secretase cleavage will allow the receptor still to signal through the membrane-anchored C-terminal fragment (mCTF) in addition to the full-length receptor. Both cleavage events are blocked when the primary shedding performed by ADAM proteases is inhibited leading the receptor to signal only through the canonical full-length receptor. The wild-type receptor in turn is able to signal through the canonical full-length receptor, the mCTF and the released soluble intracellular domain (ICD).
B: Western analysis of WM-266-4 cells expressing the indicated TYRO3 variants and treated with or without the gamma-secretase inhibitor GSI IX. The full-length receptor and the different cleavage products of TYRO3 are indicated.
C: Western analysis of the autophosphorylation of TYRO3 in WM-266-4 cells expressing the indicated TYRO3 variants.
D: Western analysis of the phosphorylation status of TYRO3 downstream effector STAT3 in WM-266-4 transfectants.
E: Confocal microscopy analysis of TYRO3 in WM-266-4 cells expressing the indicated V5-tagged variants of TYRO3. V5 signal is shown in red and DAPI-stained nuclei in blue. Nuclear localization is presented as Pearson correlation coefficient of TYRO3-V5 co-localizing with DAPI within the cells. For statistical testing, the non-parametric Kruskal-Wallis ANOVA was utilized. The post hoc analyses were conducted with the Mann-Whitney U test and the resulting P-values were corrected with the method of Benjamini, Krieger and Yekutieli. One dot represents one cell and the horizontal line the median value.
F: The workflow of interactome, phosphoproteome, proteome and transcriptome data acquisition from WM-266-4 transfectants.

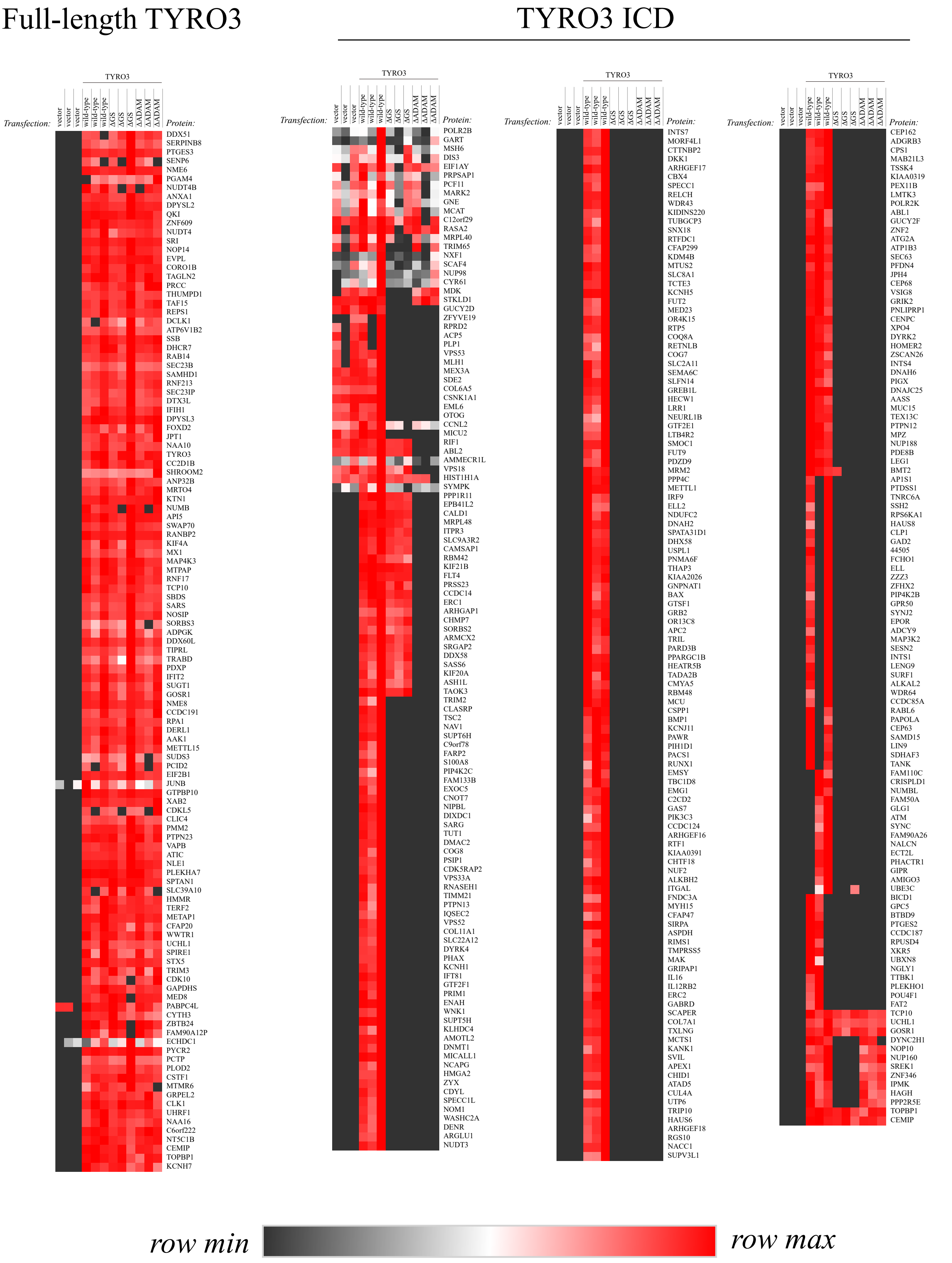

Figure S16. Interactome of full-length TYRO3 and cleaved TYRO3 ICD. The TYRO3 precipitates of WM-266-4 transfectants were analyzed with mass spectrometry. Differential expression analysis was conducted to discover the proteins co-precipitating with the full-length TYRO3 and the cleaved TYRO3 ICD. Values are presented in a relative scale. For the original values, please see Table S6. ICD: intracellular domain.

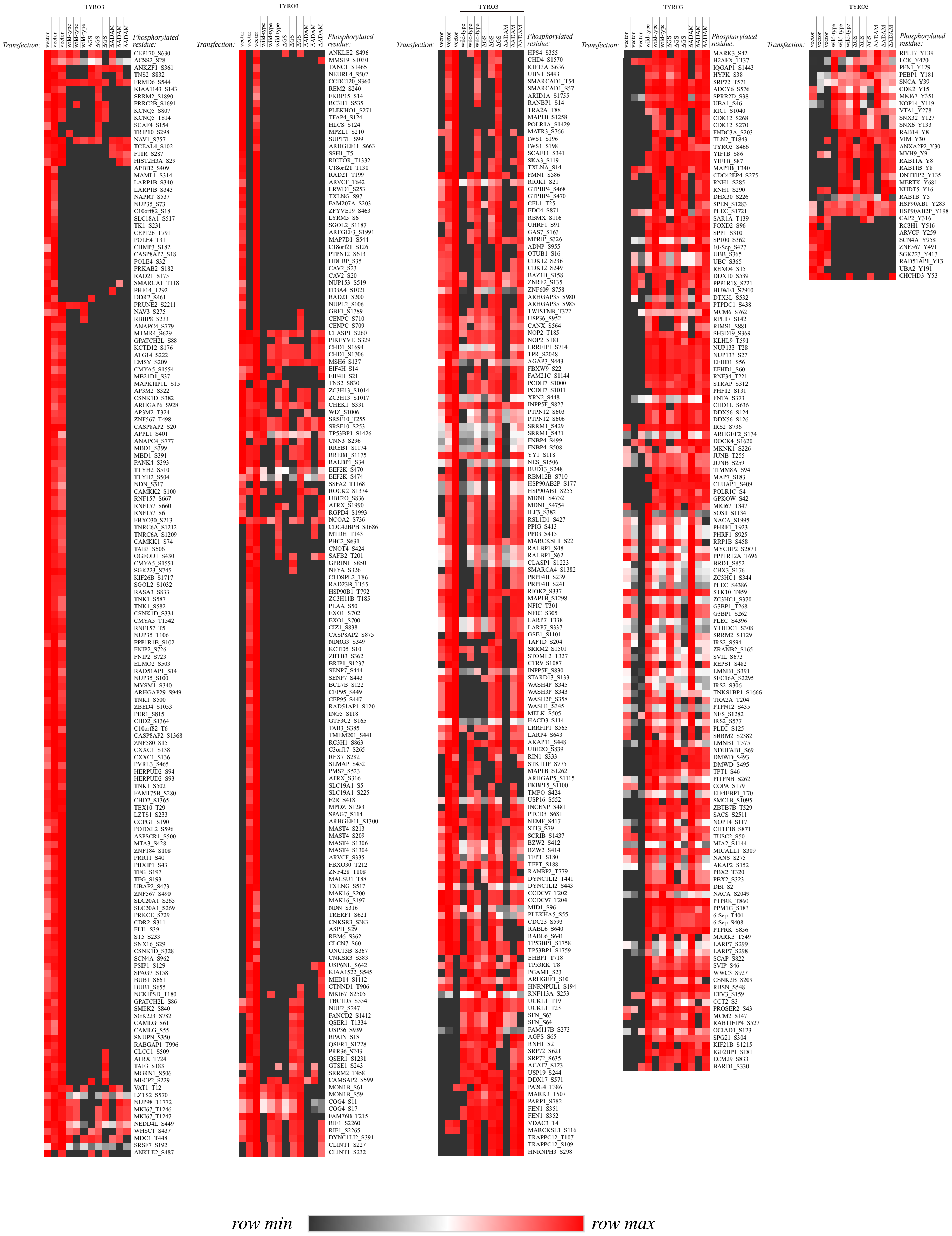

Figure S17. Differentially expressed phosphoproteome of full-length TYRO3. The phosphoproteome of WM-266-4 transfectants was acquired with mass spectrometry. Differential expression analysis was conducted to discover the differentially phosphorylated residues associated with the signaling of full-length TYRO3. Values are presented in a relative scale. For the original values, please see Table S7.

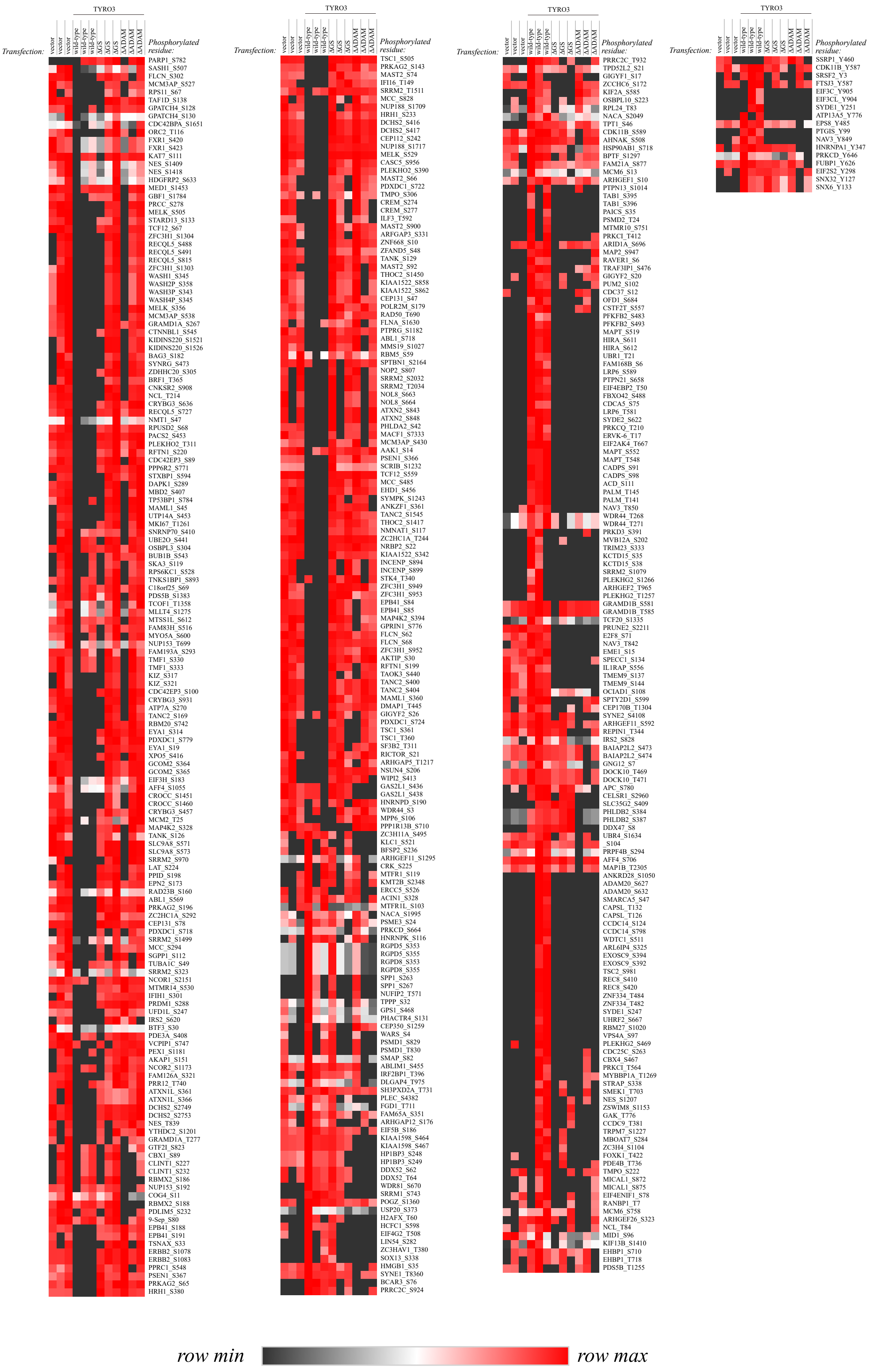

Figure S18. Differentially expressed phosphoproteome of cleaved TYRO3 ICD. The phosphoproteome of WM-266-4 transfectants was acquired through mass spectrometry. Differential expression analysis was conducted to discover the differentially phosphorylated residues associated with the signaling of TYRO3 ICD. Values are presented in a relative scale. For the original values, please see Table S7. ICD: intracellular domain.

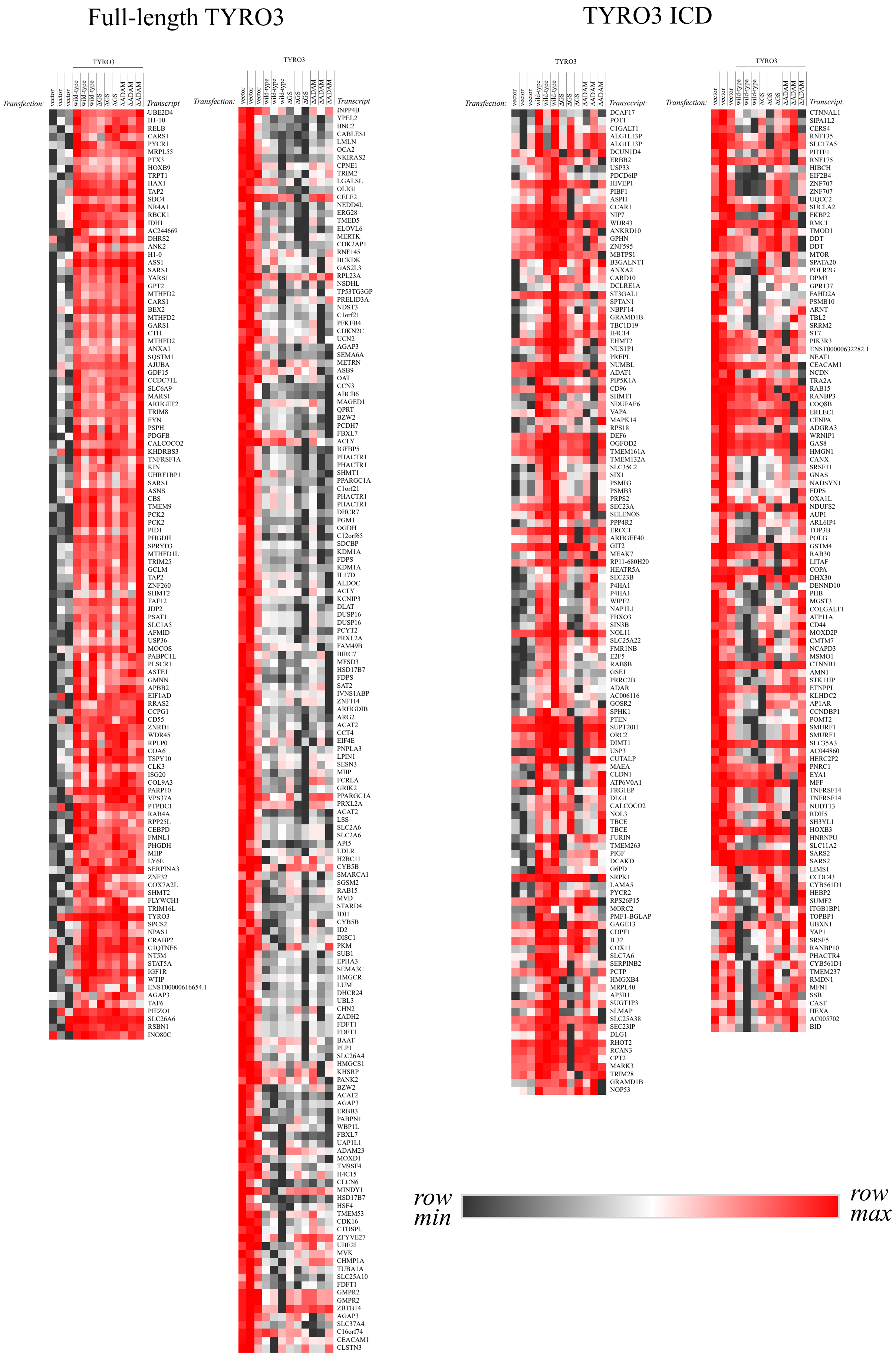

Figure S19. Differentially expressed transcriptome of full-length TYRO3 and cleaved TYRO3 ICD. The mRNA extracts of WM-266-4 transfectants were sequenced with Illumina HiSeq. Differential expression analysis was conducted to discover the differentially expressed transcripts associating with full-length TYRO3 and the TYRO3 ICD. The values are presented in relative scale. For the original values, please see Table S8. ICD: intracellular domain.

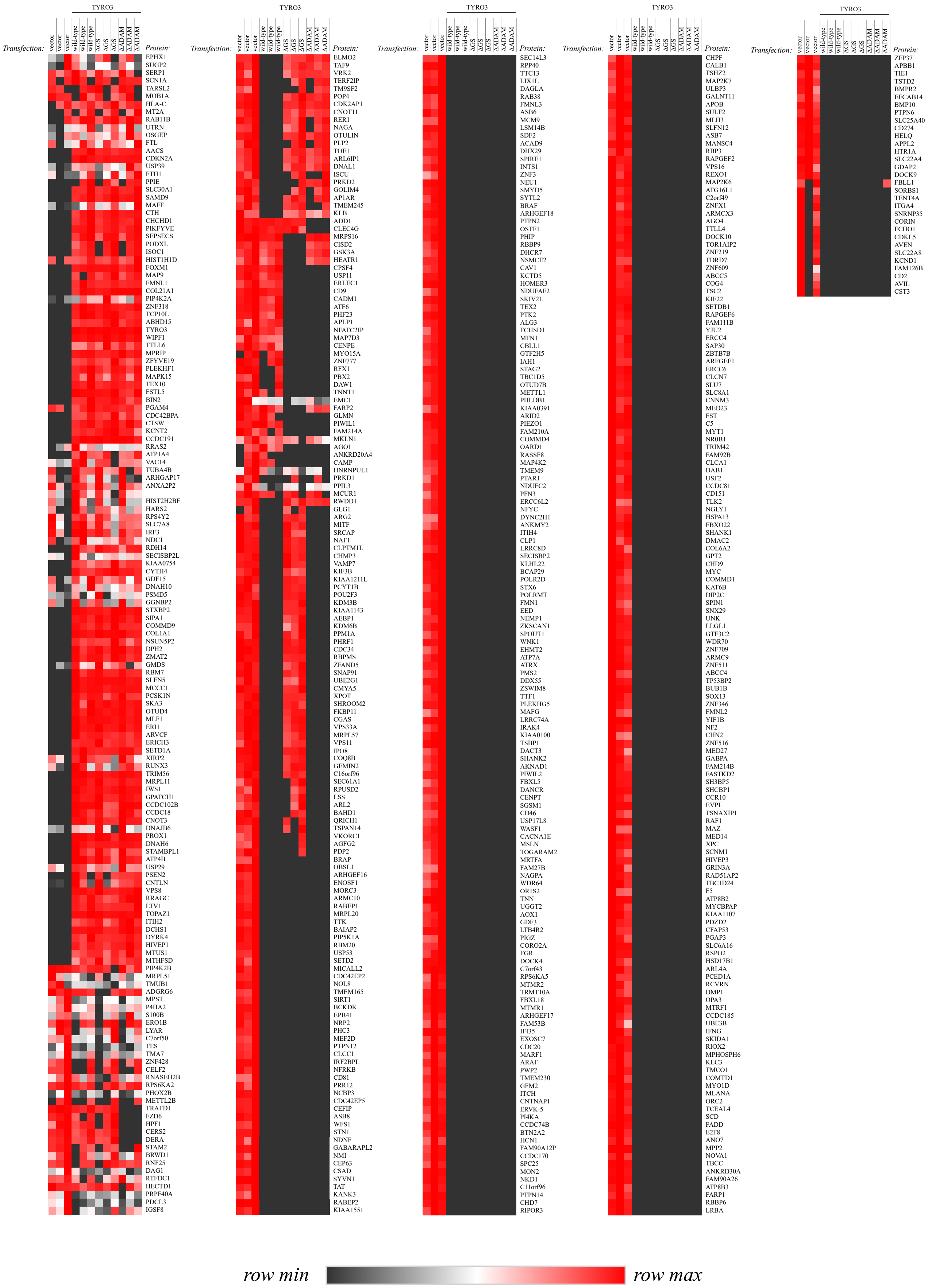

Figure S20. Differentially expressed proteome of full-length TYRO3. The proteome of WM-266-4 transfectants was acquired with mass spectrometry. Differential expression analysis was conducted to discover the differentially expressed proteins associated with the signaling of full-length TYRO3. Values are presented in a relative scale. For the original values, please see Table S9.

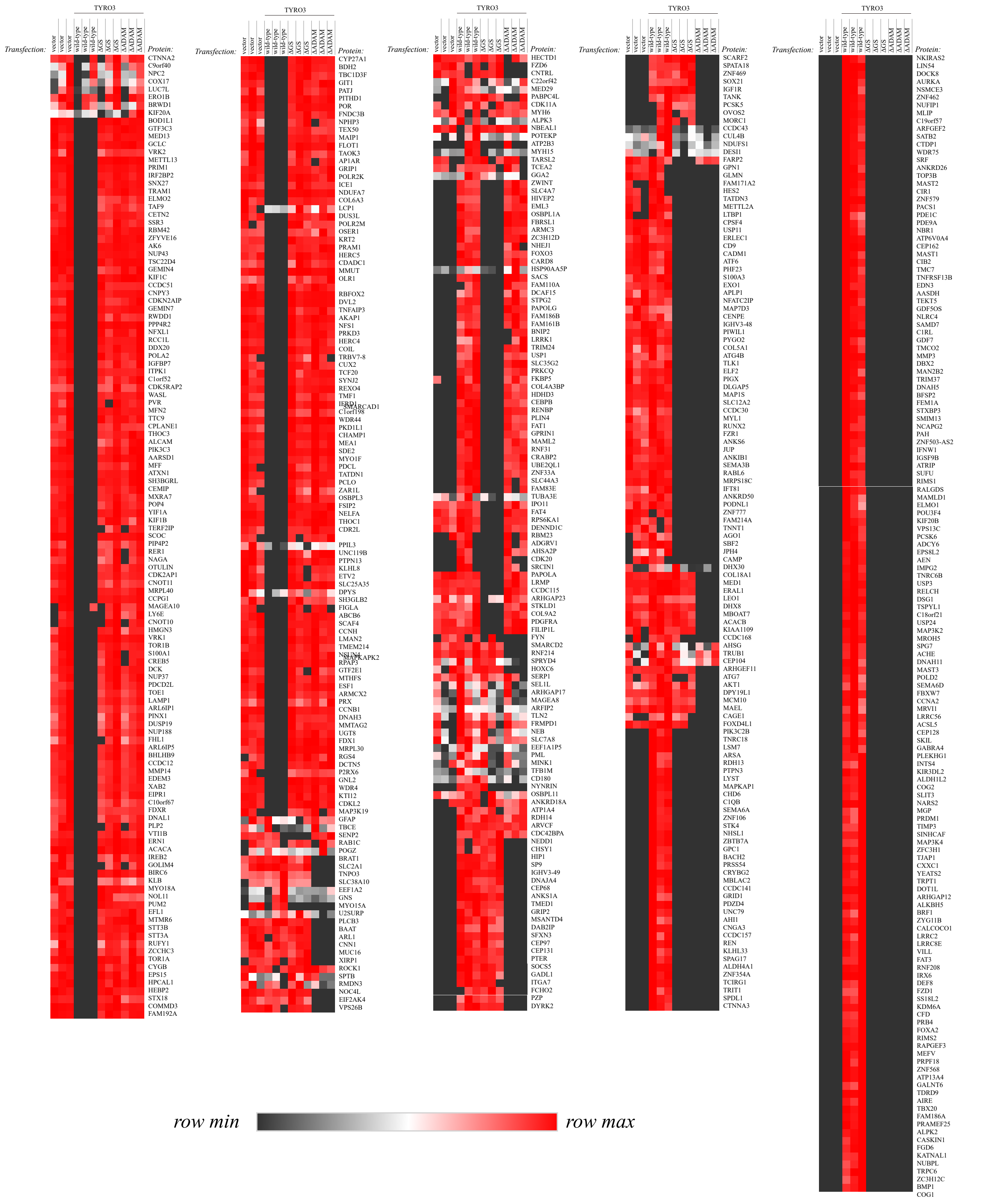

Figure S21. Differentially expressed proteome of cleaved TYRO3 ICD. The proteome of WM-266-4 transfectants was acquired through mass spectrometry. Differential expression analysis was conducted to discover the differentially expressed proteins associated with the signaling of TYRO3 ICD. Values are presented in a relative scale. For the original values, please see Table S9. ICD: intracellular domain.

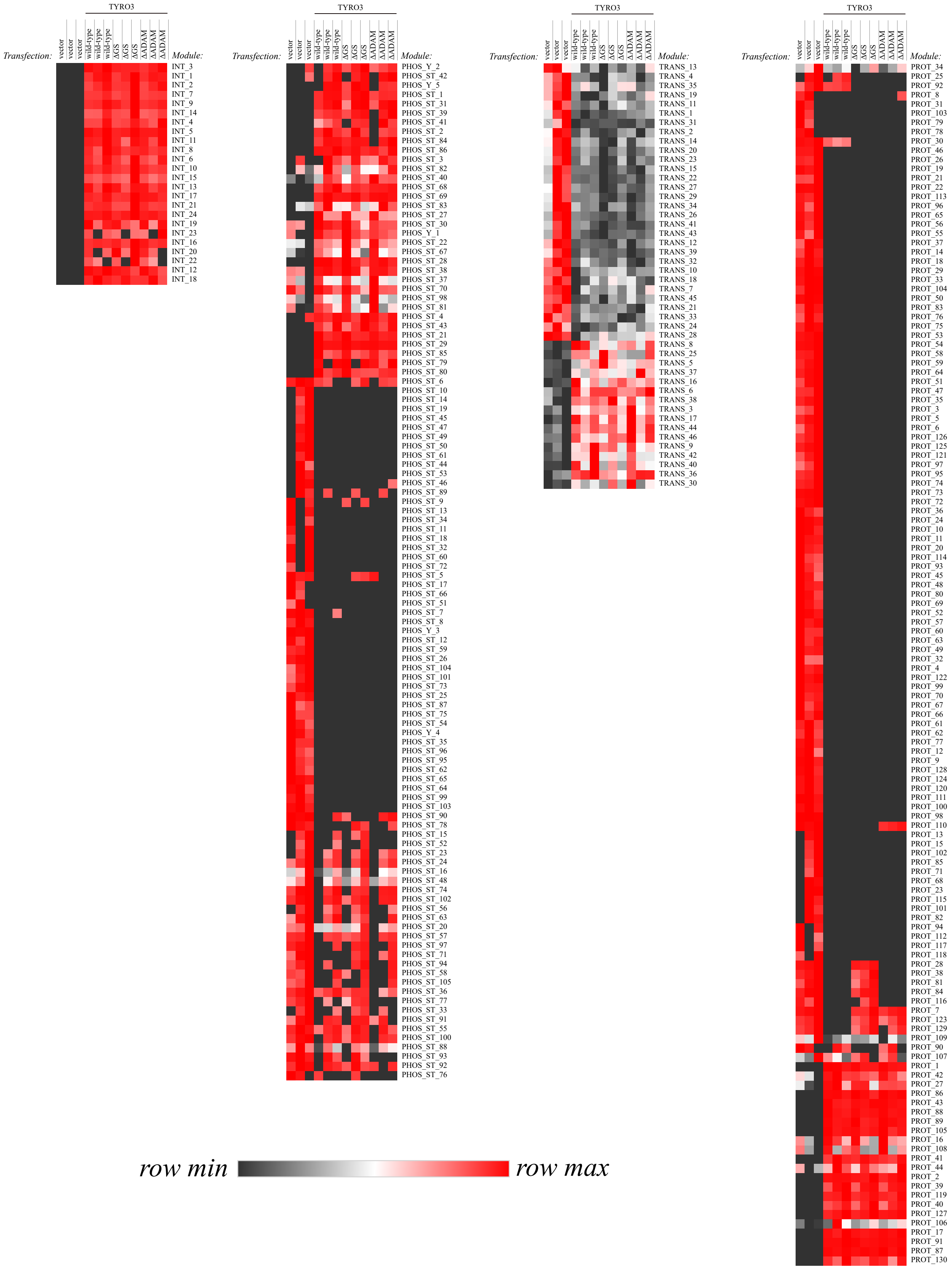

Figure S22. Network modules of full-length TYRO3. The median expression of the network modules inferred with DMPA from the multi-omics data of full-length TYRO3-associated signaling in WM-266-4 transfectants. The presented values are in relative scale. For the original values, please see Table S11.

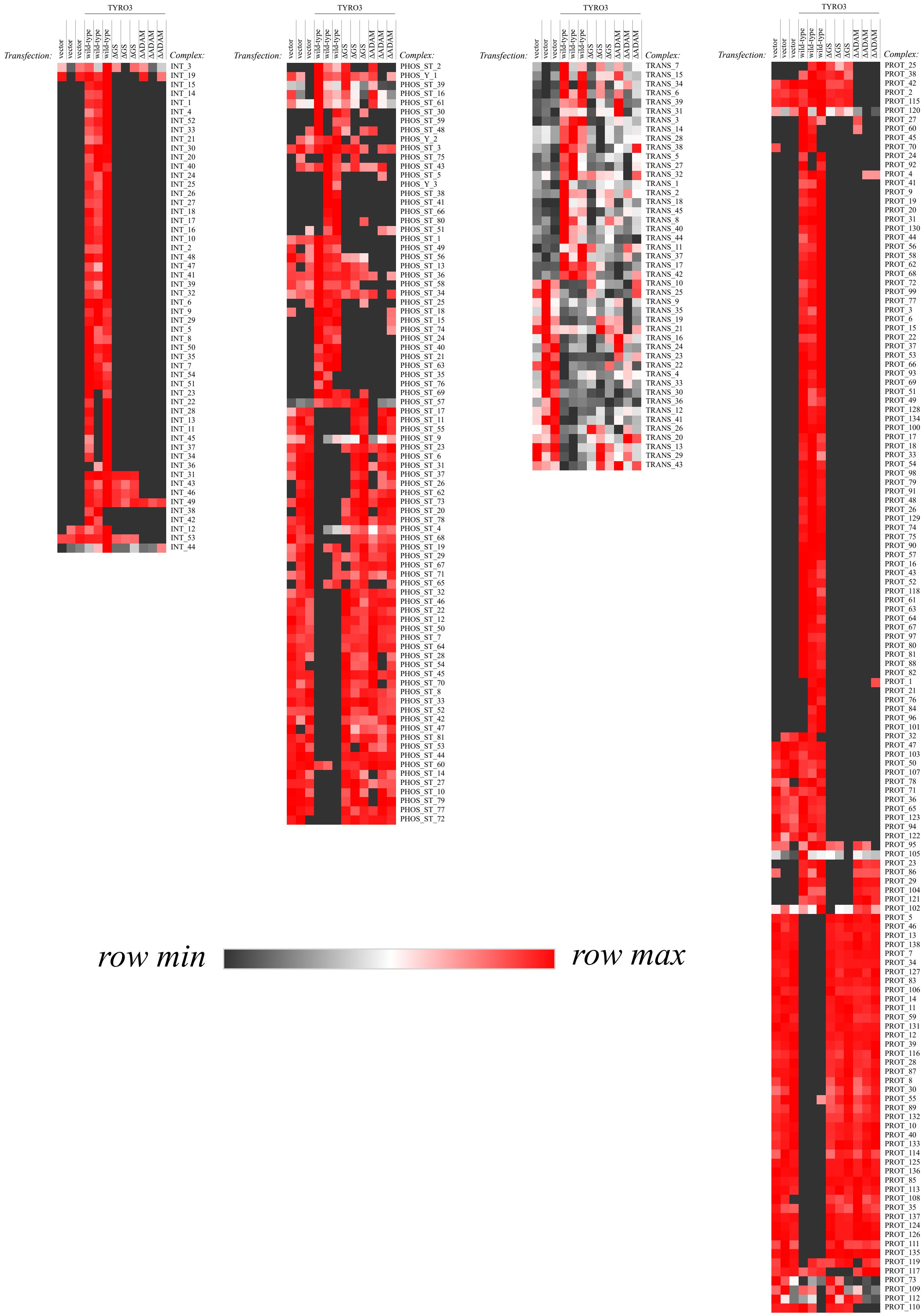

Figure S23. Network modules of cleaved TYRO3 ICD. The median expression of the network modules inferred with DMPA from the multi-omics data of cleaved TYRO3 ICD-associated signaling in WM-266-4 transfectants. The presented values are in relative scale. For the original values, please see Table S11. ICD: intracellular domain.

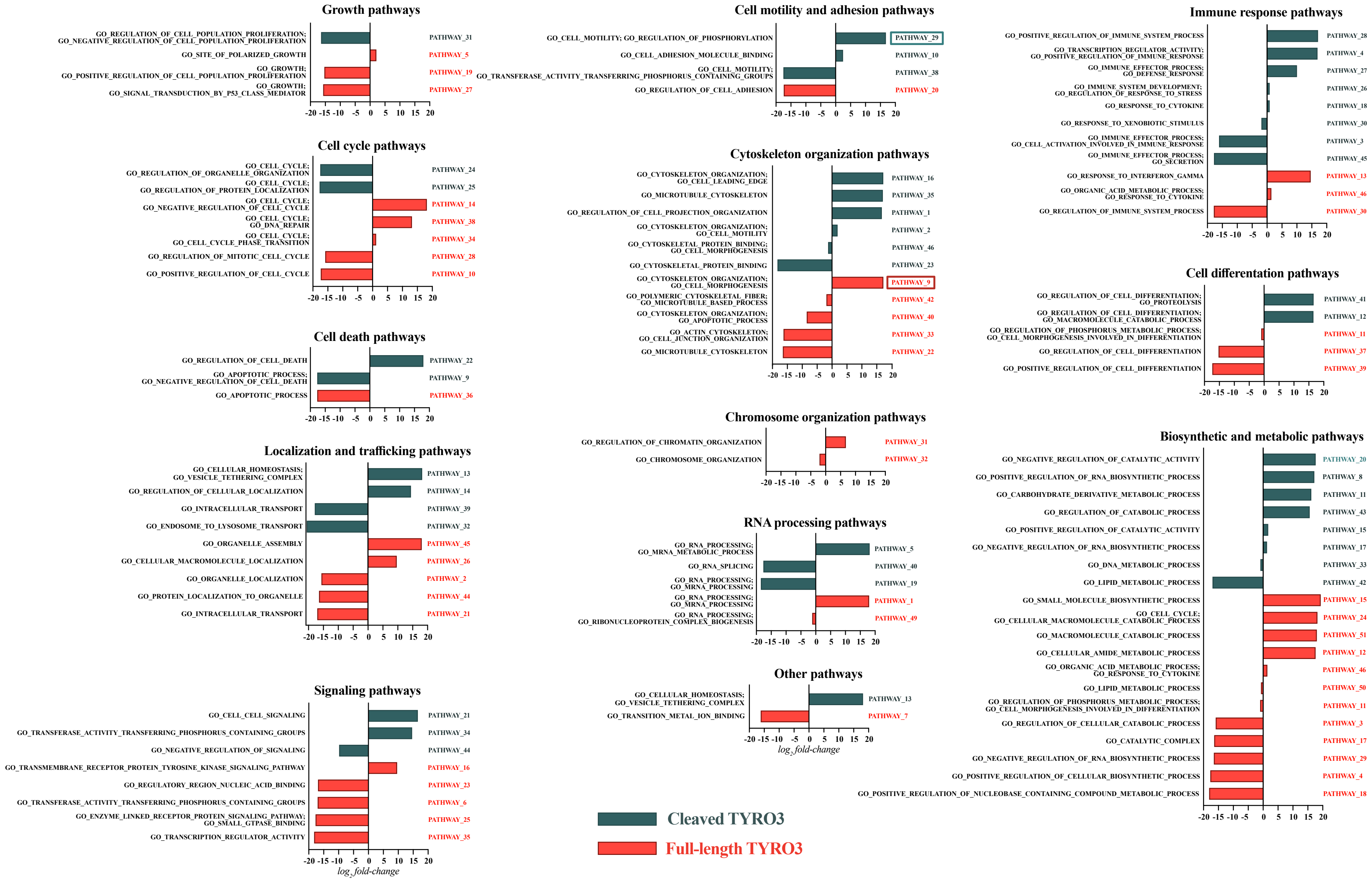

Figure S24. The functional categorization of the full-length and cleaved TYRO3 ICD pathways inferred with DMPA. The predicted function of the multi-omics pathways of the full-length and ICD of TYRO3 inferred with the de novo multi-omics pathway analysis. The bars represent the median pseudolog2 fold change of all the proteins, transcripts and phosphorylated residues in the pathway against the control condition. See Tables S1 and S2 for further details on the pathways and Table S10 for further details on the function prediction.

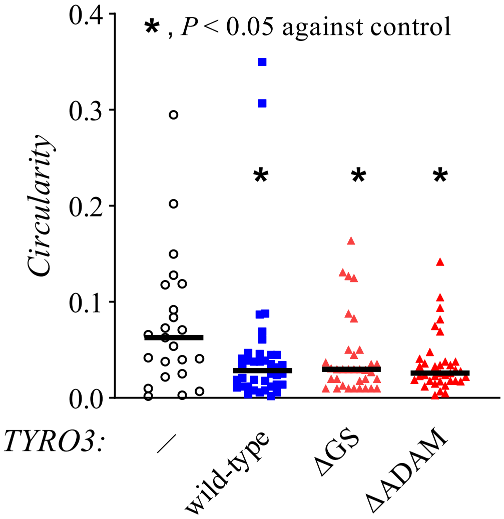

Figure S25. **Morphology of WM-266-4 transfectants.** Two-dimensional morphological analysis of WM-266-4 transfectants. Circularity of the cells was measured from confocal images taken in plane with the plasma membrane with MorphoLibJ plugin of ImageJ. For statistical testing, the non-parametric Kruskal-Wallis ANOVA was utilized. The post hoc analyses were conducted with the Mann-Whitney U test and the resulting P-values were corrected with the method of Benjamini, Krieger and Yekutieli. One dot represents the morphology of one cell and the horizontal line the median value. ΔADAM, ADAM cleavage mutant; ΔGS, gamma-secretase cleavage mutant.

Pseudocode of the DMPA algorithm

For each omics dataset:

Network module inference:

Calculate pairwise combination scores and initial edges list:

For each feature in omics dataset

Calculate pairwise (Spearman rank) correlation score

If parameter 3 == 1 (true)

Take absolute value of correlation score

End

End

Rank correlation scores by feature

Adjust correlation scores based on rank to value range from 0-1 (highest correlation score is assigned 1)

Find duplicate correlation scores:

If correlation score of feature a (i) == correlation score of feature a (j)

adjusted correlation score(i) and adjusted correlation score(j) = average(adjusted correlation score([i j]))

End

For each feature in omics dataset:

Calculate pairwise stoichiometry score:

Check how many non-missing values you have in the expression values both features of the feature pair

Check how many missing values you have in the expression values of both features of the feature pair

Check how many combinations of missing and non-missing values you have in the expression values of features of the feature pair

If more than 1 non-missing value in the same samples(1:n) in both features of the feature pair:

If parameter 4 == 1 (true)

Calculate zero-inflated version of the stoichiometry score:

If median(feature(i,non-missing)) >= median(feature(j,non-missing)) == TRUE

Stoichiometry score(i,j) = Interquartile range ([feature(i,non-missing)/feature(j,non-missing) repeat(1, amount of both missing) repeat(100000, amount of one missing one non-missing)])

Else

Calculate non-zero-inflated version of the stoichiometry score:

If median(feature(i,non-missing)) >= median(feature(j,non-missing)) == TRUE

Stoichiometry score(i,j) = Interquartile range (feature(i,1:n)/feature(j,1:n))

Else

Stoichiometry score(i,j) = Interquartile range (feature(j,1:n)/feature(i,1:n))

End

End

Else

Stoichiometry score(i,j) = 100000

End

Rank stoichiometry scores by feature

Adjust stoichiometry scores based on descending rank to value range from 0-1 (lowest stoichiometry score is assigned 1)

Find duplicate stoichiometry scores:

If stoichiometry score of feature a (i) == stoichiometry score of feature a (j)

adjusted stoichiometry score(i) and adjusted stoichiometry score (j) = average(adjusted stoichiometry score([i j]))

End

Derive the combined score by element-wise multiplication of pairwise adjusted correlation and stoichiometry scores

Derive initial edge list:

Create initial list from feature pairs with combined scores >= 1-(1/number_of_features)

Calculate how many times each feature appears in the initial list and sort it from lowest to highest value

While The number of times a feature which index is defined by the parameter S value appears in the sorted list < the value of the C parameter

Reduce the cut-off of by 1-(1/number_of_features)

Create a new initial edge list from feature pairs with combined scores >= new cut-off

Calculate how many times each feature appears in the initial list and sort it from lowest to highest value

End

Filter edges out from the edge list where the absolute difference between the combined score (i,j) and combined score (j,i) is higher than the threshold: parameter 10 * 1/number_of_features

Find maximally scoring 3-node cliques from the initial edges list

Remove duplicate edges in the new edges list derived from the maximally scoring 3-node cliques

Combine 3-feature cliques into larger modules:

While two modules with 2 common feature exist in the module list

Join module x and module y with 2 common features

End

Remove duplicate modules from the list

Join small modules with one common feature (optional):

While two modules with 1 common feature with maximum size defined by parameter 7 and score defined by parameter 8 exist in the module list

Join small module x and module y with 1 common features

End

Remove duplicate modules from the list

Join modules with 2 common features:

While two modules with 2 common feature exist in the module list

Join module x and module y with 2 common features

End

Remove duplicate modules from the list

Remove features from the remaining 3-feature cliques that are common with larger modules

Join small modules with one common feature (optional):

While two modules with 1 common feature with combined size defined by parameter 9 exist in the module list

Join small module x and module y with 1 common feature

End

Remove duplicate modules from the list

Return list of modules, list of module indices

End

Network module combination to pathways:

For each omics dataset:

Create a median matrix from list of module indices and expression values in the original dataset file

End

Combine the median matrices from different omics layers

Use the combined median matrix and the network module inference function to derive pathways

Return list of pathways

End

Figure S15B

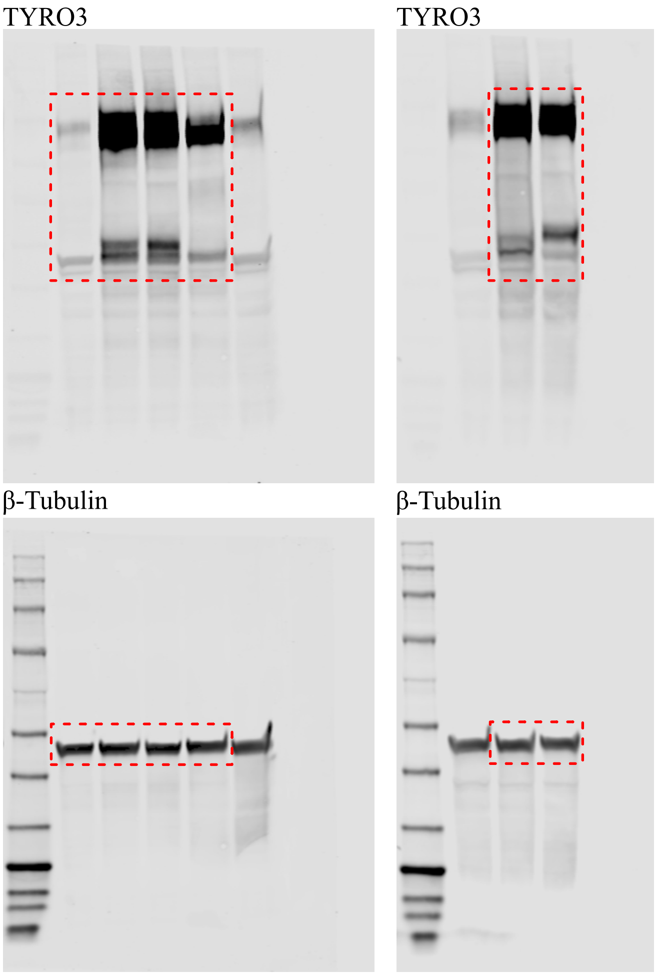

Figure S15C

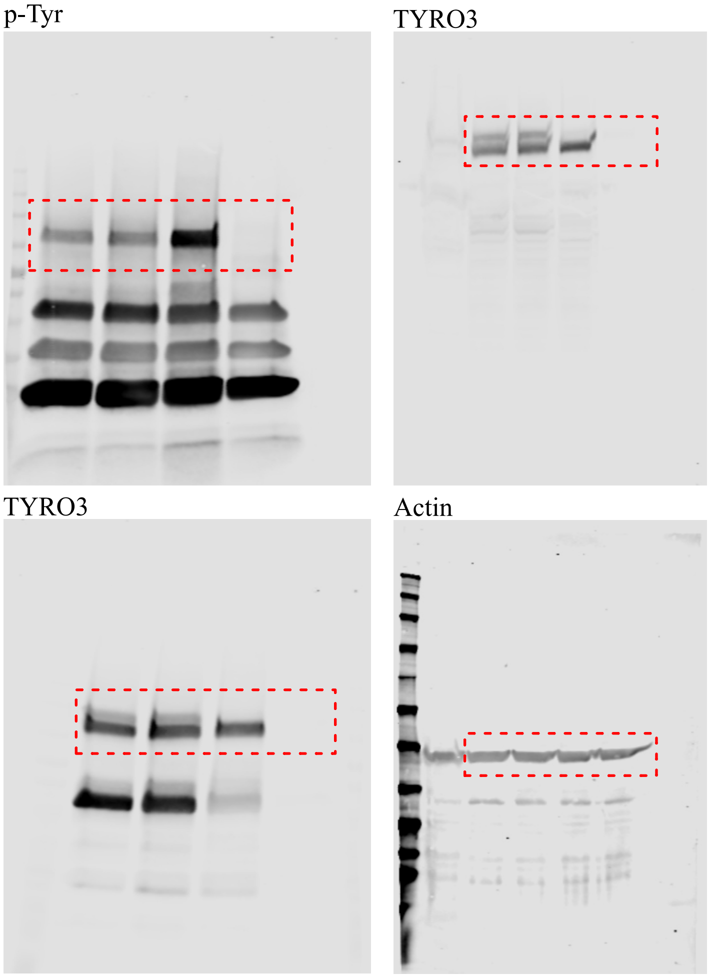

Figure S15D

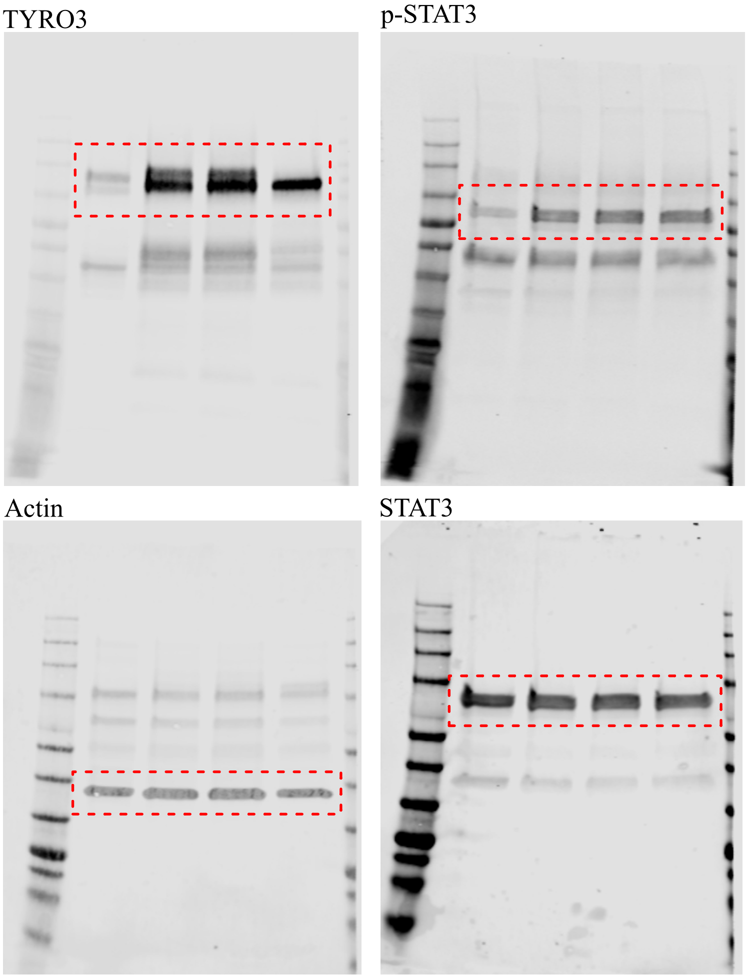

Supplement: Supplemental Data [file mmc1.pdf]
